# Coordinated satellite, aircraft, and ground-based observations of a large transient methane release

**DOI:** 10.1073/pnas.2603595123

**Published:** 2026-09-01

**Authors:** Tai-Long He, Daniel J. Varon, Shobha Kondragunta, Xinrong Ren, Mark D. Cohen, Brian J. Carroll, Nathan Malarich, Jeff Peischl, Tobias A. de Jong, Jelmar Gerritsen, Joannes D. Maasakkers, Daniel H. Cusworth, Riley M. Duren, Steven S. Brown, Carsten Warneke, Colm Sweeney, Phillip Stratton, Alan Brewer, Sunil Baidar, Ezra J. T. Levin

**Affiliations:** ^a^ Institute for Data, Systems, and Society, Massachusetts Institute of Technology, Cambridge, MA 02139; ^b^ Department of Aeronautics and Astronautics, Massachusetts Institute of Technology, Cambridge, MA 02139; ^c^ Center for Satellite Applications and Research, National Environmental Satellite, Data, and Information Service, National Oceanic and Atmospheric Administration, College Park, MD 20740; ^d^ Air Resources Laboratory, National Oceanic and Atmospheric Administration, College Park, MD 20740; ^e^ Cooperative Institute for Research in Environmental Sciences, University of Colorado Boulder, Boulder, CO 80309; ^f^ Chemical Sciences Laboratory, National Oceanic and Atmospheric Administration, Boulder, CO 80305; ^g^ Global Monitoring Laboratory, National Oceanic and Atmospheric Administration, Boulder, CO 80309; ^h^ Netherlands Institute for Space Research, Leiden 2333 CA, The Netherlands; ^i^Carbon Mapper, Inc., Pasadena, CA 91101; ^j^ Department of Atmospheric and Oceanic Science/Cooperative Institute for Satellite Earth System Studies, University of Maryland, College Park, MD 20742; ^k^ Energy Institute, Colorado State University, Fort Collins, CO 80523; ^l^ Department of Systems Engineering, Colorado State University, Fort Collins, CO 80523

**Keywords:** methane, satellites, remote sensing, release experiment, pipeline blowdown

## Abstract

We present the results of a Very Large Methane Release (VLMR) experiment evaluating methane retrievals from the Geostationary Operational Environmental Satellites (GOES) Advanced Baseline Imagers (ABIs) and multiple low-Earth-orbit satellite instruments with high point-source detection thresholds. The experiment coordinated observations of a US gas pipeline blowdown with nine satellites, an aircraft, and a truck-based mobile laboratory. We used the GOES-16, -18, and -19 ABIs with revisits every 10 min to 7 s to quantify release magnitude and associated uncertainty. Best methane retrieval precision of 7% was achieved in the 30-s mesoscale scan modes averaged to 5 min, yielding an estimated methane plume detection threshold of 15 to 30 t h^−1^ per m s^−1^ of wind. GOES detected total emissions of 370 ± 30 t over 42 to 63 min from two release points. Source rate and mass estimates are broadly consistent across measurement platforms, but the total detected release mass is ~25% lower than that reported by the operator based on pipeline volume and pressure. This discrepancy may reflect late-stage emissions below satellite detection thresholds and indicates a potential low bias in satellite estimates of total emissions from large transient releases. Coordinated field experiments such as VLMR can complement existing controlled-release satellite evaluation programs by providing a framework to validate observations of very large methane point sources.

## Significance

Methane is a potent greenhouse gas, and emissions from oil and gas infrastructure are a major mitigation target. Satellites are increasingly used to detect and quantify these emissions, but evaluating their estimates of releases remains challenging. In this study, we analyze a US gas pipeline blowdown in New Mexico using coordinated observations from nine satellites, an aircraft, and a truck-based mobile laboratory. Our experiment provides a rare opportunity to evaluate the accuracy of satellite-based observations of large, short-lived methane point sources. We show that geostationary satellites can continuously track and quantify such releases, with emission estimates consistent with other platforms and expectations from pipeline pressure and volume. Our results build confidence in satellite observations to monitor extreme methane releases worldwide.

Methane is a potent greenhouse gas whose concentration is rapidly increasing in the atmosphere, posing a major challenge for climate change mitigation. A small number of high-emitting point sources account for a disproportionate fraction of total methane emissions (1, 2). Satellite observations have become an important tool for detecting and quantifying these sources to identify effective mitigation opportunities (3). A growing constellation of low-Earth-orbit (LEO) satellite instruments provides a wide array of complementary capabilities for monitoring methane point sources, ranging from high-resolution imagers for targeted detection of relatively small plumes (0.1 to 1 t h^−1^) to wide-swath instruments with lower sensitivity but greater spatial and temporal coverage enabling detection of large releases (>10 t h^−1^) over broad areas (4, 5, 6). More recently, methane point-source monitoring has extended to geostationary orbit (GEO), enabling continuous hemispheric observations every few minutes and the detection of extreme, transient releases on the order of tens to hundreds of tons per hour (7, 8).

Despite this progress, substantial uncertainties remain in satellite detection thresholds and quantification accuracy, particularly for high-emitting sources. Previous studies have evaluated detection and quantification performance indirectly, based on the population statistics of detected plumes (2, 5, 9) or analysis of synthetic plume data (10, 11). Detection and quantification performance has also been directly evaluated using controlled release experiments with metered ground-truth source rates, which are increasingly common but have so far been limited to sources below 10 t h^−1^ due to cost and safety constraints (12–14). These experiments provide valuable benchmarks for instruments with detection thresholds of ~1 t h^−1^ or lower but are not well-suited to evaluating higher-threshold instruments (~10 t h^−1^ or larger) such as the Tropospheric Monitoring Instrument (TROPOMI) in LEO and the GOES Advanced Baseline Imager (ABI) in GEO.

Here we present the results of a Very Large Methane Release (VLMR) experiment evaluating the ability of such instruments to detect and quantify emissions. The experiment was conducted on 8 October 2024 in New Mexico, United States, and involved coordinated observations of a planned gas pipeline blowdown with a suite of ground, aerial, and satellite platforms. We quantify the resulting emissions using observations from three GOES ABIs, six LEO instruments, an aircraft, and a truck-based mobile laboratory. The GOES ABI observations from three vantage points in GEO provide measurement frequencies of 10 min down to 7 s, allowing estimation of time-dependent plume mass, emission rate, and associated uncertainties. The LEO instruments provide additional snapshots of the evolving plumes, and the aircraft and ground-based platforms provide independent estimates of source rate along with detailed information on the timing of the blowdown, local meteorology, and in-situ methane concentrations. Estimates of total plume mass and source rate are compared across measurement platforms and benchmarked against the operator’s reported release mass based on pipeline pressure and volume.

## Experimental Setup

**Fig. 1.**
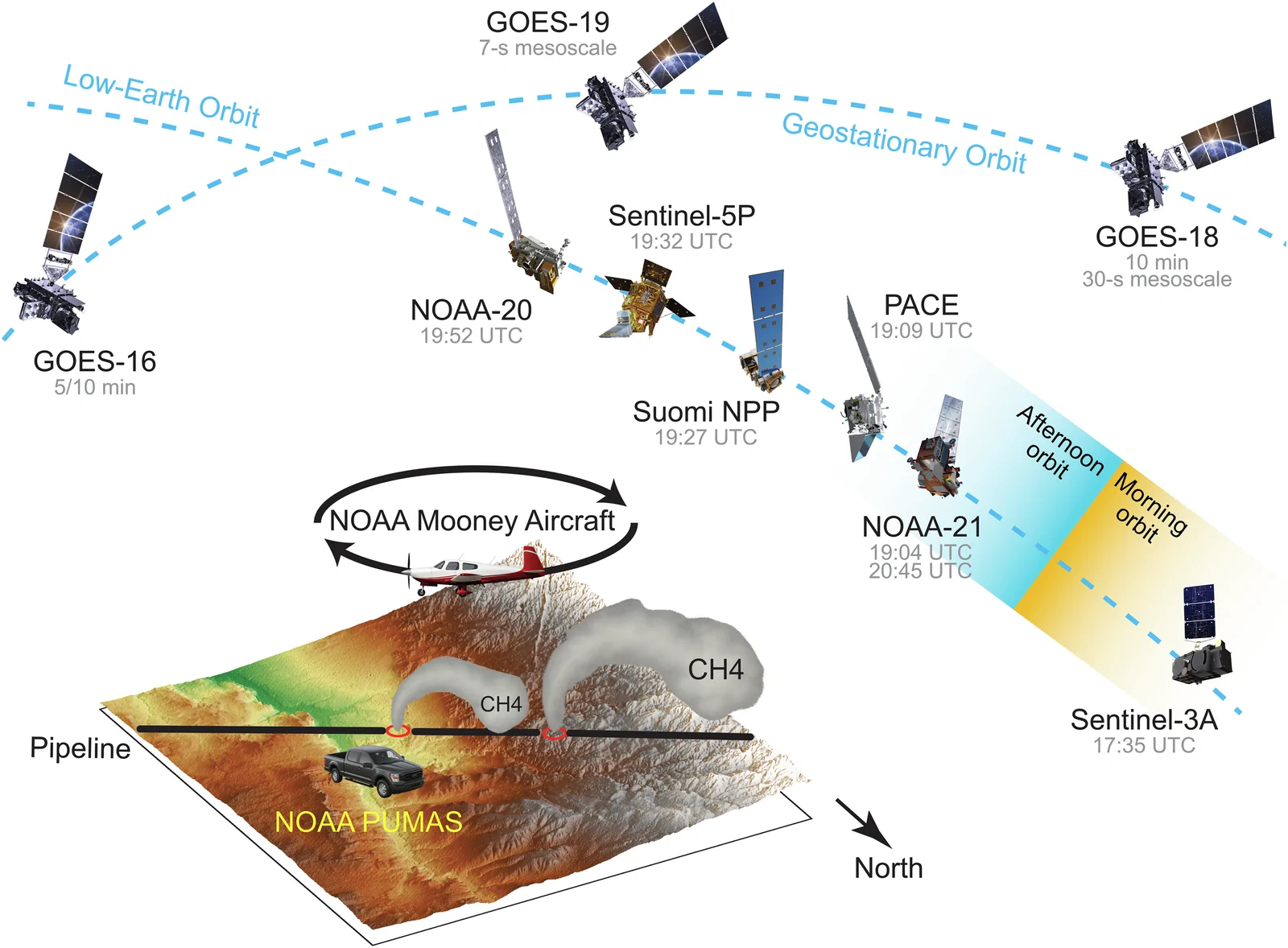
Schematic of the 8 October 2024 VLMR experiment to quantify methane (CH_4_) emissions from a planned gas pipeline blowdown in New Mexico, United States. Measurements were collected from 3 GOES satellites, six LEO satellites, a Mooney aircraft, and a truck-based mobile laboratory. GOES scan frequencies (5/10-min operational modes and targeted 7 to 30-s mesoscale modes) and LEO overpass times (UTC; LT + 6) are annotated in gray. The red circles indicate the two locations along the pipeline where the blowdown occurred.

Fig. 1 illustrates the VLMR experimental setup. The blowdown occurred at two release points along the pipeline, hereafter referred to as the eastern and western sites. Emissions began at 17:23 UTC (LT + 6) at the western site and 17:36 UTC at the eastern site. The pipeline operator reported total emissions of 27.5 million standard cubic feet (scf) of natural gas, corresponding to 506 t of methane at 96% methane content. The plumes were observed from space by the GOES-16, -18, and -19 ABIs; TROPOMI; the Visible Infrared Imaging Radiometer Suite (VIIRS) on the Suomi-NPP, NOAA-20, and NOAA-21 satellites; the PACE Ocean Color Instrument (OCI); and the Sentinel-3A Sea and Land Surface Temperature Radiometer (SLSTR). In situ measurements were from a Mooney aircraft equipped with a gas concentration analyzer and NOAA’s Pick-Up Based Mobile Atmospheric Sounder (PUMAS) platform.

At the time of the VLMR experiment, GOES-16 and -18 were in their East and West positions at 75.2°W and 137.2°W, respectively, and GOES-19 was undergoing commissioning at 90°W. In addition to the operational 5 to 10-min scan modes from GOES-16 and -18, we scheduled 30-s targeted mesoscale scans with GOES-18, and the commissioning of GOES-19 provided an opportunity to test an experimental 7-s mesoscale scan mode. The mesoscale scans targeted a ~1,000 × 1,000-km^2^ domain centered on the pipeline release points. In total, we analyze five continuous data streams from the three GOES satellites. The LEO satellites observed the releases primarily in the local afternoon (19:04–20:45 UTC), with one morning pass by Sentinel-3A (17:35 UTC). More detailed information on the measurement platforms and scan modes is provided in *Materials and Methods*.

## Release Mass Estimates

**Fig. 2.**
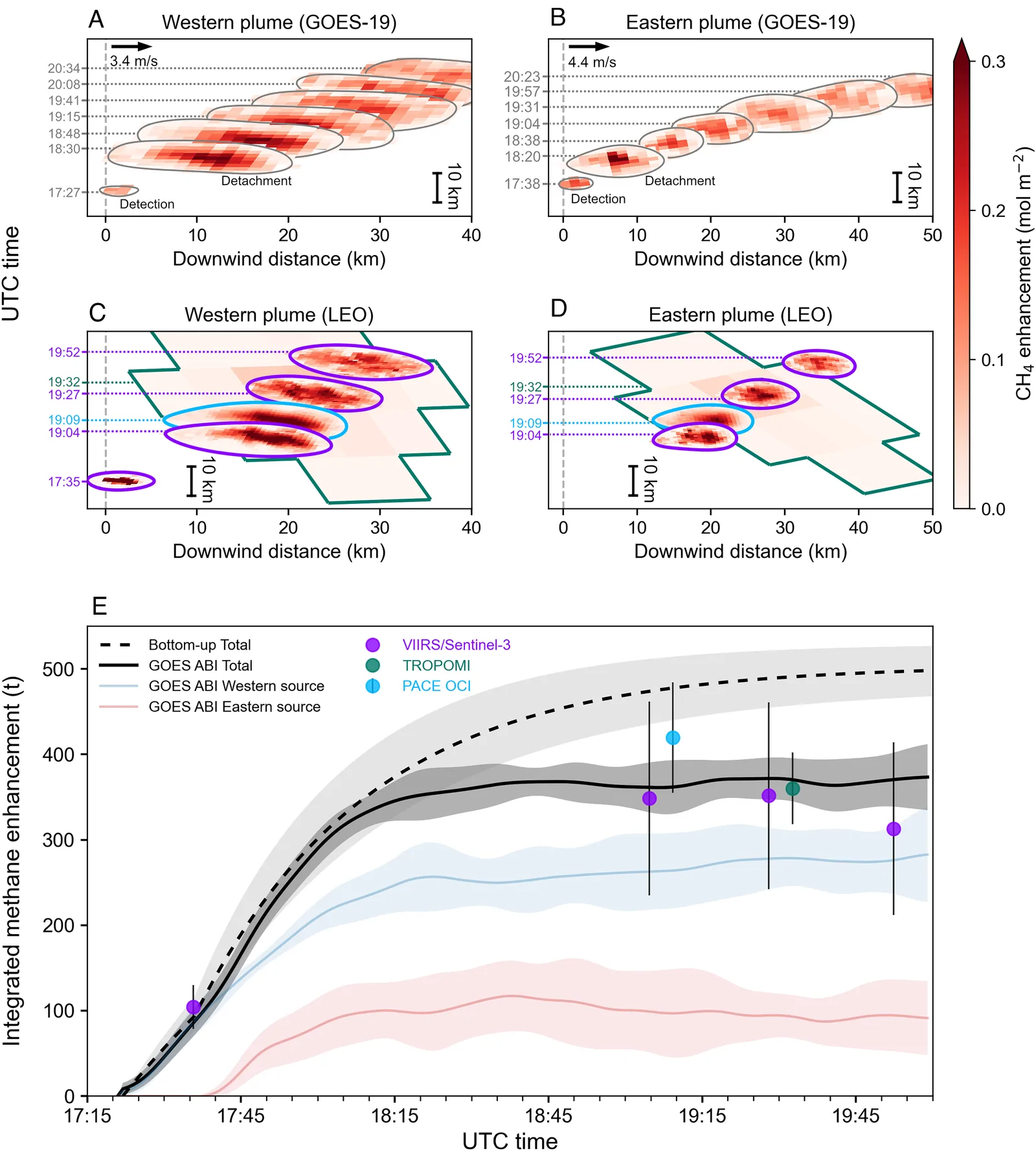
Methane plume evolution observed by GEO and LEO satellites during the pipeline blowdown. (*A* and *B*) Sequential GOES-19 ABI retrievals showing masked methane enhancements downwind of the two sources, with plume mask contours (smoothed for visualization) in gray. Plumes are vertically offset in time to reduce overlap for visualization. First detection and detachment snapshots are labeled. Local wind speed estimates from the High-Resolution Rapid Refresh (HRRR) product are shown *Inset*. (*C* and *D*) Same as (*A* and *B*) but for LEO satellite observations at different overpass times. Plume contour lines and overpass times are color-coded by instrument consistently with the markers in panel (*E*). The latest NOAA-21 pass, at 20:45 UTC (*SI Appendix*, Fig. S2), is not shown. (*E*) Time series of total methane plume mass, expressed as integrated methane enhancement (IME). Solid lines show the mean IME across the five GOES ABI scan modes interpolated to 1 min and smoothed with a 9-min moving average (*Materials and Methods*). The blue and red lines are for the western and eastern sources, respectively, and the black line is for their sum. Dark gray shading indicates ±1σ uncertainty ranges derived from the five GOES ABI scan modes. The circular markers represent IME derived from LEO satellites for the sum of both plumes. The dashed line shows bottom–up estimates based on pipeline pressure and volume reported by the operators, modeling the release as an exponential decay process. Light gray shading shows the propagated uncertainty in the bottom–up estimates from the release duration and total release mass (*Materials and Methods*).

Fig. 2 presents snapshot retrievals of methane enhancements from the three GOES and six LEO satellites, together with observational (top–down) and modeled (bottom–up) estimates of plume mass over time. The plumes were first detected in the GOES mesoscale scans at 17:27 UTC and 17:38 UTC. Movie S1 shows their full temporal evolution as observed from GEO. The eastern plume detached from its source at 18:20 UTC and the western plume at 18:30 UTC, corresponding to observed emission durations of 42 to 63 min, respectively (Fig. 2 *A* and *B*). The LEO instruments captured the releases at a sequence of overpass times and show broad agreement with the GOES retrievals in plume magnitude, location, and extent (Fig. 2 *C* and *D*), despite substantial differences in pixel size, viewing geometry, and retrieval precision. VIIRS, Sentinel-3 SLSTR, and PACE OCI, with nadir pixel resolutions of 500 m to 1 km, clearly resolve plume structure. Methane enhancements are strongly diluted within the coarser TROPOMI pixels (~5.5 × 7 km^2^ at nadir), softening plume edges and broadening the apparent footprints. GOES ABI’s high temporal resolution and intermediate spatial resolution (~2 km at nadir) captures sufficient structural detail to track the spatiotemporal evolution of the plumes.

The observed plume mass, expressed in Fig. 2*E* as IME (15), exhibits an initial growth phase followed by stabilization as the plumes detach from their sources and are advected downwind, consistent with previous GEO satellite observations of large transient releases (8). LEO satellites are limited to discrete overpass times, and in this case captured the release near the onset and after plume detachment but missed much of the growth phase. This illustrates a general limitation of LEO platforms for quantifying transient methane sources, whose episodic nature may prevent satellites with fixed overpass times from detecting actual release locations or observing specific stages of plume development. GOES ABI infers a total detectable methane mass of 370 ± 30 t, with 260 ± 40 t from the western source and 100 ± 50 t from the eastern source, while LEO estimates for total mass range from 310 ± 100 t to 420 ± 65 t (individual source breakdown shown in *SI Appendix*, Fig. S13), generally within 5 to 15% of the GOES multi-instrument mean and consistent within uncertainties.

Satellite IME estimates are considerably lower than the pipeline operator’s reported total release of 506 t: 27% lower for GOES ABI and 17 to 39% lower for the LEO instruments, with the most precise multispectral retrieval from PACE OCI yielding the smallest mass bias (*SI Appendix*, *Text S5*). The dashed line in Fig. 2*E* models the blowdown releases as two exponential decay processes (*Materials and Methods*), with a total duration of 3 h for the pipeline to reach atmospheric pressure, as reported by the operators. This bottom–up estimate agrees closely with the IME inferred from GOES ABI and Sentinel-3 SLSTR during the plume growth phase (~17:22–18:00 UTC), but diverges during the stabilization phase, when the satellite-observed mass begins to fall below the modeled trajectory. This discrepancy may reflect the satellites’ failure to capture emissions below their detection thresholds, which occur later in the release. It may also reflect weak, dispersed enhancements that were not captured by the plume masks. While the satellites effectively track the initial methane pulses over the first hour of the blowdown, the detected mass should be interpreted as a lower bound on total emissions. In this case, it underestimates the total by about 25%.

## Source Rate Estimates

**Fig. 3.**
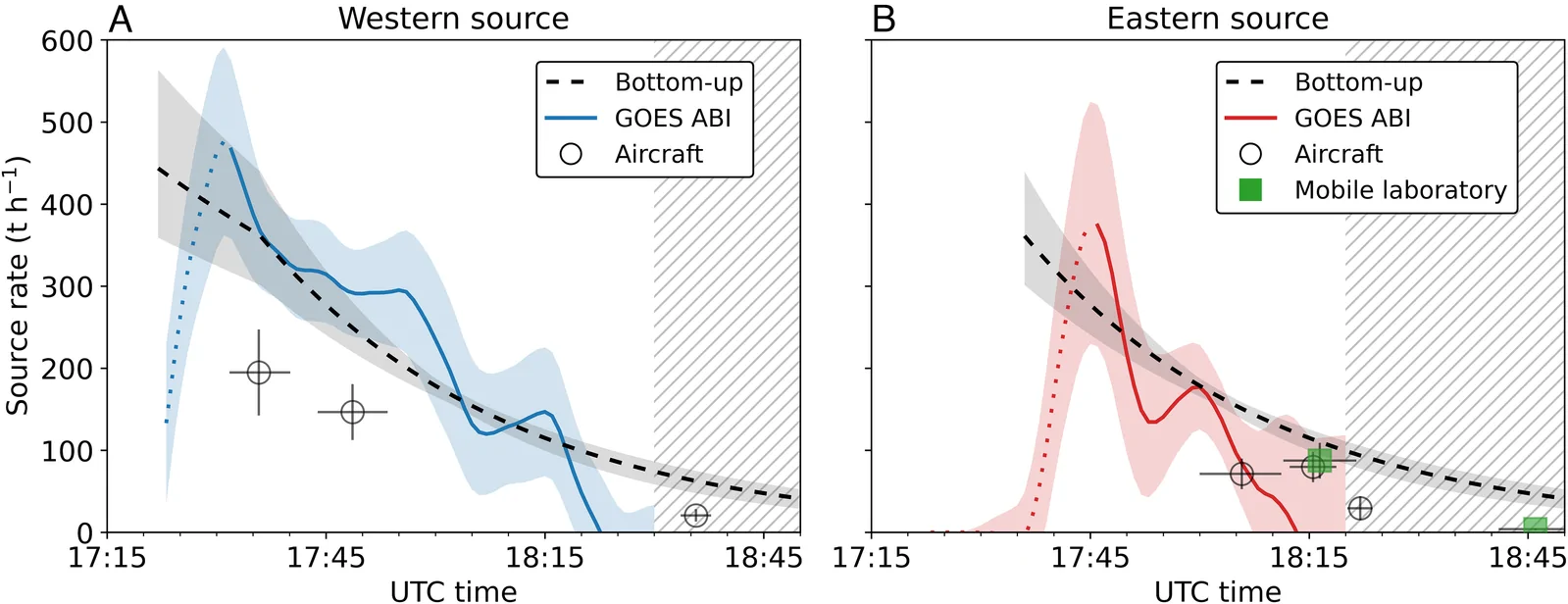
Instantaneous source rates for the (*A*) western and (*B*) eastern sources inferred from GOES ABI and in situ measurements. ABI source rates are defined as the time derivative of IME (Fig. 2). Dotted line segments mark periods of initial mass accumulation when instantaneous fluxes are not well defined (see text). Solid lines show the mean source rate across the five GOES ABI retrievals, blue/red shaded regions indicate ±1σ uncertainties, and the hatched regions mark the period after plume detachments. Dashed lines represent bottom–up estimates based on total release mass (~500 t methane) and blowdown duration (3 h) as reported by the pipeline operators. Light gray shading shows uncertainty in the bottom–up estimates from release duration and total release mass (*Materials and Methods*). Circular and square markers show source-rate estimates derived from in situ measurements using aircraft and ground-based mass-balance calculations.

Quantifying source rates from single-pass plume images typically requires information on the local wind speed, which is highly uncertain (16). Here, the fine sampling frequency of GOES ABI enables a wind-free source-rate retrieval based on temporal changes in plume mass, which capture variability in emissions that single-pass retrievals cannot easily resolve (7). Fig. 3 shows instantaneous source rates inferred from ABI retrievals as the time derivative of IME for each plume from initial release to detachment. We find that the source rate peaked at about 500 t h^−1^ for the western source and 360 t h^−1^ for the eastern source within 10 min of the onset of the releases, and then declined over the following ~30 to 60 min. The initial growth in source rate (dotted lines in Fig. 3) likely reflects nonlinear absorption effects from strong subpixel concentration gradients early in the release. Increasing the methane concentration within a dense subpixel plume would produce only small changes in the pixel-average radiance, biasing retrievals low. This effect would weaken as the plume diffuses across one or more full pixels.

Our GOES ABI-derived source rates agree with the bottom–up exponential model within uncertainties for both release points. They show higher peak values and lower minima, dropping below zero as the emission signals approach the noise level of the retrieval shortly before plume detachment. Agreement between satellite-derived and in situ estimates is also within uncertainties for the eastern source. For the western source, however, satellite-derived and bottom–up source-rate estimates are roughly a factor of two higher than inferred from the first two aircraft spirals. In situ estimates are generally lower than expected from the bottom–up model, with low biases of 40 to 70% for the western source and 20 to 90% for the eastern source.

We tested whether this low bias could reflect saturation of the Picarro analyzer outside its nominal operating range [0 to 20 ppm; (17)] for the western source. The peak measured concentration during the VLMR experiment was 226 ppm, far outside the nominal range, but laboratory tests using a 193 ppm methane standard indicate that the instrument remains accurate at these elevated concentrations (*SI Appendix*, Fig. S11). The bias is therefore more likely attributable to undersampling of the transient plume, which is not expected to be well-mixed within the boundary layer (*SI Appendix*, Fig. S12). Future VLMR experiments could employ flight patterns with larger spiral radii to sample a more fully mixed plume, or multiple aircraft at different radii to better characterize plume evolution over the course of the release. The comparatively close agreement between GOES ABI and the bottom–up model highlights the value of geostationary satellite observations for quantifying methane plume mass and instantaneous source rate.

The limitations of the bottom–up model must also be recognized. It assumes equal partitioning of the release between the eastern and western sites, neglecting potential differences in vent geometry and supercritical fluid dynamics within the pipeline (18). It may also overestimate late-stage source rates by neglecting cooling during blowdown, including Joule-Thomson effects that can lead to icing or hydrate formation at the vent orifice, reducing the effective mass flow (19). Future VLMR experiments could incorporate on-site measurements of pressure and temperature along the pipeline, especially near the release points, to provide better ground-truth information on source rates over time.

## Retrieval Precision and Detection Threshold

The VLMR experiment provided an opportunity to characterize GOES ABI methane retrieval precision in different scan modes. Quantifying precision as the SD of retrieved methane enhancements across scans and excluding pixels containing clouds, water, and plumes, we find best retrieval precision of 0.044 mol m^−2^ (7% of 1,930 ppb background) in the 30-s scan mode averaged to 5 min, followed by 0.052 mol m^−2^ (8% of background) in the 7-s scan mode averaged to 5 min. Precision for the other ABI scan modes is 10 to 12%. Sensitivity tests using different averaging windows show that 5-min averaging provides the best balance between noise reduction and plume signal preservation for both the 7-s and 30-s scans (*SI Appendix*, Figs. S14 and S15). Time-averaged mesoscale scans therefore provide a higher-precision complement to hemispheric scan modes for geostationary methane plume monitoring.

Finally, we use GOES ABI’s fine temporal sampling to assess the plume detection threshold Q_min_ (t h^−1^) for the VLMR scene. In the finest-precision 30-s mesoscale scan mode, the plumes are first detected at the single-pixel level with column enhancements of 0.07 to 0.08 mol m^−2^ over 3.35-km pixels, reflecting a single-pixel methane mass of ~12 t for initial detection. The steady-state emission rate required to sustain such an enhancement against horizontal transport across the pixel is Q_min_ = 15 t h^−1^ per m s^−1^ of wind, which we take as a lower bound on GOES ABI’s detection threshold. We also estimate Q_min_ using the heuristic equation introduced by Jacob et al. (20):

[1] $$Q_{\min}\;=\;M_{{\mathrm{CH}}_{4}}\cdot U\cdot L\cdot q\cdot \sigma ,$$

where $M_{{\mathrm{CH}}_{4}}$ is the molar mass of methane, U (km h^−1^) is the wind speed, pixel size L = 3.35 km, and q is the number of SD above the background noise level σ required for single-pixel detection (q = 2 by convention). Under 4 m s^−1^ winds (Fig. 2) and assuming 7% precision for σ, this yields a minimum detectable source rate of 110 t h^−1^ (27 t h^−1^ per m s^−1^), consistent with the uncertainty ranges in Fig. 3. We therefore estimate a detection threshold of 15 to 30 t h^−1^ per m s^−1^ of wind for GOES ABI for the VLMR scene. This is similar to the detection threshold of 30 to 50 t h^−1^ estimated by Zhou et al. (8) for the higher-resolution Meteosat Flexible Combined Imager (FCI) geostationary satellite instrument under 3 to 4 m s^−1^ winds (~10 to 15 t h^−1^ per m s^−1^ of wind).

## Conclusions

We used a VLMR experiment to evaluate satellite capabilities for quantifying large transient methane emissions through coordinated multiplatform observations of a planned US gas pipeline blowdown. Continuous GOES ABI measurements from three instruments in five scan modes captured the evolution of the release with down to subminute temporal resolution and detected total methane emissions of 370 ± 30 t from two release points. The blowdown was also observed by six LEO satellite instruments, a Mooney aircraft, and a truck-based mobile laboratory. Estimates of total release mass are consistent across satellite platforms but ~25% lower than the operator’s reported value based on pipeline pressure and volume. The satellite observations effectively track the initial methane pulses downwind but may miss weaker emissions below instrument detection thresholds later in the release. In situ mass-balance analysis underestimates early source rates by ~50%, reflecting the difficulty of sampling rapidly evolving, heterogeneous plumes from transient point sources. Our results confirm the capability of satellite instruments to quantify large transient methane releases from both low-Earth and geostationary orbit, while also revealing a potential low bias in total detected emissions. Expanding VLMR experiments across a broader range of observing conditions would further refine satellite performance characterization and improve understanding of the contribution of large transient releases to regional and global methane budgets.

## Materials and Methods

### GOES ABI GEO Satellite Data.

GOES ABI is a multispectral scanning radiometer with 16 channels in the visible (VIS), near-infrared (NIR), and infrared (IR) that support a wide range of weather, ocean, and environmental monitoring applications. Pixel resolution is 0.5 km (VIS), 1 km (NIR), or 2 km (IR) at equatorial nadir. ABI has three operational scan modes that provide coverage of the full hemispheric disk every 10 min, the contiguous US (CONUS) every 5 min, and targeted mesoscale sectors (~1,000 × 1,000-km^2^ domain) every 30 to 60 s. GOES-16 operated as GOES-East at 75.2°W from 2016 to 2025, covering the eastern US, Atlantic Ocean, and part of western Africa. It was superseded by GOES-19 in April 2025 and now serves as backup satellite at 104.7°W. GOES-18 currently serves as GOES-West at 137.2°W and covers the western US, Pacific Ocean, and Alaska.

We use the Level-1b (L1b) radiances and Level-2 (L2) cloud mask data from a total of five scan modes across all three ABIs to perform methane retrievals for the VLMR pipeline releases with sampling frequencies from 10 min down to 7 s.

GOES ABI can detect large transient methane releases in its shortwave-infrared (SWIR) bands at 1.6 µm and 2.25 µm via multiband–multipass (MBMP) methane retrievals (7). The retrievals compare images from the methane-sensitive 2.25-µm band with reference images from the 1.6-µm band and previous scans. To retrieve methane column concentrations, we define for each image pixel a band ratio.

[2] $$r\;=\;\frac{c*R_{2.25}}{R_{1.6}},$$

where $R_{1.6}$ and $R_{2.25}$ represent the measured radiances in the 1.6-μm and 2.25-μm bands and c is a scaling factor to address differences in scene-wide mean brightness between the two bands. r is converted to methane column enhancement (mol m^−2^) using a lookup-table derived from radiative transfer calculations. For each resulting retrieval image, we construct a plume-free reference retrieval image from previous (preblowdown) scans and subtract it to better isolate methane plumes from surface artifacts (see *SI Appendix*, *Text S2* for additional details).

To account for differences in observational scan times, each of the five GOES ABI data streams was first interpolated to a 1-min temporal resolution and then smoothed using a 9-min moving average. The IME values and source rates plotted in Figs. 2 and 3 were computed as the mean across the five streams, with error bars representing the SD. Sensitivity tests using different averaging windows (5 to 15 min) show that the results are robust to this choice (*SI Appendix*, Fig. S13).

### LEO Satellite Data.

#### TROPOMI.

TROPOMI is a high-spectral-resolution spectrometer on the Sentinel-5P satellite (21). Its local overpass time is around 13:30 local time (LT) and its methane retrieval product has a nadir pixel resolution of 5.5 × 7 km^2^. TROPOMI’s fine 0.25 nm spectral resolution enables high-quality methane retrievals with <1% precision (22) and detection of large methane point sources (5). We use the operational TROPOMI L2 total methane column data product (23) to quantify the total methane mass released during the VLMR experiment (*SI Appendix*, *Text S3*). TROPOMI’s fine retrieval precision makes it an effective baseline for evaluating methane plume mass estimates from less sensitive multispectral retrievals.

#### VIIRS and SLSTR.

De Jong et al. (24) demonstrated the use of multiple multispectral satellite radiometers for subdaily monitoring of methane point sources, including VIIRS on the Suomi-NPP, NOAA-20, and NOAA-21 satellites, and SLSTR on the Sentinel-3A and -3B satellites (25). The three VIIRS instruments fly in sun-synchronous afternoon orbits with observations around 13:30 LT, while the SLSTR instruments observe in the morning around 10:00 LT. The methane retrieval algorithm for VIIRS and SLSTR uses the difference of the ratios of reflectance between target and reference images in the 1.6- and 2.25-µm SWIR bands (*SI Appendix*, *Text S4*). The nadir spatial resolution of the methane retrieval is 750 m for VIIRS and 500 m for SLSTR. At these resolutions, facility-level methane plumes can be detected, and the subdaily monitoring frequency combining all instruments makes it possible to capture short-lived emission events such as pipeline blowdowns.

#### OCI.

OCI is an imaging radiometer on NASA’s Plankton, Aerosol, Cloud, ocean Ecosystem (PACE) satellite, launched in February 2024. OCI provides SWIR measurements at 1.6, 2.1, and 2.3 µm with 1.2-km spatial resolution. The 2.1-µm band exhibits weak methane absorption and is spectrally closer than the 1.6-µm band to the methane-sensitive 2.3-µm band, making it a more effective methane-free reference channel for MBMP retrievals. Details about the OCI methane retrieval algorithm are given in *SI Appendix*, *Text S5*.

#### Aircraft data.

NOAA Global Monitoring Laboratory performed ~1 km radius spiral profiles around the two release sites and flew cross-plume transects ~25 km downwind of the western site (*SI Appendix*, Fig. S5) using a ChampionX Mooney aircraft instrumented with a Picarro 2401-m gas concentration analyzer. Methane mixing ratios were measured every 2.5 s and interpolated to 1 Hz, with estimated uncertainties of ±2 ppb. We use these measurements to estimate emission rates Q (kg h^−1^) for both pipeline release points using a mass-balance approach:

[3] $$Q\;=\;\int \left[\frac{M_{{\mathrm{CH}}_{4}}}{M_{\mathrm{air}}}\cdot \rho_{\mathrm{air}}\cdot 10^{-9}\right]\cdot \left[C\left(t\right)\;-\;C_{b}\right]\cdot U_{p}\left(t\right)\cdot dA,$$

Here C(t) is the measured methane concentration, Cb is the background concentration, Up (t) is the wind velocity component perpendicular to the plume cross-section, dA is a differential element of the plume cross-sectional area with vertical extent defined by the helix pitch for spirals (26) or the boundary layer height for cross-plume transects (27). The factor $\frac{M_{{\mathrm{CH}}_{4}}}{M_{\mathrm{air}}}\cdot \rho_{\mathrm{air}}\cdot 10^{-9}$ converts methane mixing ratios from nmol mol^−1^ to mass concentration, where M_CH4_ and M_air_ are the molecular weights of methane and dry air, respectively, and ρ_air_ is the air density (*SI Appendix*, *Text S6*). Wind speed and direction used in the mass-balance calculations and shown in Fig. 2 are taken from the hourly HRRR (28) reanalysis at 10 m above ground level.

#### Ground-based measurements.

NOAA Chemical Sciences Laboratory performed ground-based measurements with the PUMAS platform to estimate emission rates from plume transects downwind of the eastern source. PUMAS is a motion-stabilized Doppler lidar (29), augmented for this experiment with an Aeris mid-infrared absorption analyzer measuring methane and ethane at 1 Hz.

We calculate the mass flux for 10-min transects across the eastern plume ~9 km downwind of the source location using the mass-balance approach of Eq. **3**, assuming the plume is vertically well-mixed in one vertical layer with a cross-sectional area dA. The equation variables are derived from PUMAS measurements of methane mixing ratio, mixed-layer height, and 3D lidar wind fields. The calculated mass flux is assigned to an earlier pipeline release time based on estimated transport time between pipeline and transect (*SI Appendix*, *Text S7*).

### Bottom–Up Exponential Decay Model.

We model the methane release from the pipeline as an exponential decay process describing the depressurization of the pipeline from initial to atmospheric pressure. Based on the operator’s reported total gas release of 27.5 million scf (506 t methane) and release duration of 3 h, the cumulative release methane mass at each site (MW and ME) is modeled across two emission stages as follows:

$$M_{W}\left(t\right)\;=\;\left\{\begin{array}{c} M_{W,0}\left(1\;-\;e^{-\frac{t\;-\;t_{0}}{\tau_{1}}}\right),t_{0}\;\leq \;t\;<\;t_{E},\\ M_{W}\left(t_{E}\right)\;+\;M_{W,0}^{\prime }\left(1\;-\;e^{-\frac{t\;-\;t_{E}}{\tau_{2}}}\right),t\;\geq \;t_{E,} \end{array}\right.$$

$$M_{E\left(t\right)}\;=\;\left\{\begin{array}{c} 0,t\;<\;t_{E}\\ M_{E,0}\left(1\;-\;e^{-\frac{t\;-\;t_{E}}{\tau_{2}}}\right),t\;\geq \;t_{E} \end{array}\right.,$$

where t_0_ and t_E_ are the onset times of the western and eastern releases, respectively, M_W,0_ = 506 t and M_W,0_′ are the asymptotic release masses of the western source in each stage, M_E,0_ = M_W,0_′ is the asymptotic release mass of the eastern source, and $\tau_{1}$ and $\tau_{2}$ are the decay time constants before and after the eastern valve opened. The reported total release duration of 3 h implies $\tau_{1}$ = 60 min and $\tau_{2}$ = 30 min. Fitting the decay model to the GOES ABI IME data yields similar values of $\tau_{1}$ = 62 min and $\tau_{2}$ = 31 min, implying a total duration of 186 min to achieve atmospheric pressure.

The uncertainty associated with the bottom–up estimates in Figs. 2 and 3 is estimated by combining a ±5% uncertainty in the operator-reported total release mass with release durations of 150, 180, and 210 min (spanning 5τ to 7τ). The shaded envelope spans the range across all combinations of the upper and lower bounds of these two parameters.

## Supplementary Material

Appendix 01 (PDF)

Movie S1. Video footage of the methane plumes observed by GOES ABI.

## Data Availability

The NOAA GOES Level-1b radiances and Level-2 cloud mask products are publicly available via the AWS Open Data Registry (https://registry.opendata.aws/noaa-goes). The GOES ABI retrieved methane total columns have been deposited in Zenodo (https://doi.org/10.5281/zenodo.18443915) (30). TROPOMI Level-2 methane retrievals and Sentinel-3 Level-1 radiances are available from the Copernicus data services (https://www.copernicus.eu). VIIRS and PACE-OCI Level-1 radiances are available from NASA Earthdata (https://earthdata.nasa.gov). All other data are included in the manuscript and/or supporting information.
