# Supplementary material for: Coordinated satellite, aircraft, and ground-based observations of a large transient methane release
Source: Proc Natl Acad Sci U S A. 2026 Sep 1;123(36):e2603595123. doi: 10.1073/pnas.2603595123 (PMC13552872; doi:10.1073/pnas.2603595123)
Supplement: Supplementary file 1 [file pnas.2603595123.sapp.pdf]

PNAS

Supporting Information for

Coordinated satellite, aircraft, and ground-based observations of a large transient methane release.

Tai-Long He[1], Daniel J. Varon[1,2], Shobha Kondragunta[3], Xinrong Ren[4], Mark D. Cohen[4], Brian J. Carroll[5,6], Nathan Malarich[5,6], Jeffrey Peischl[5,7], Tobias A. de Jong[8], Jelmar Gerritsen[8], Joannes D. Maasakkers[8], Daniel H. Cusworth[9], Riley M. Duren[9], Steven S. Brown[6], Carsten Warneke[6], Colm Sweeney[7], Phillip Stratton[4,10], Alan Brewer[6], Sunil Baidar[5,6], Ezra J. T. Levin[11]

[1]Institute for Data, Systems, and Society, Massachusetts Institute of Technology, [2]Department of Aeronautics and Astronautics, Massachusetts Institute of Technology, [3]NOAA/NESDIS, [4]NOAA Air Resources Laboratory, [5]Cooperative Institute for Research in Environmental Sciences (CIRES), University of Colorado Boulder, [6]NOAA Chemical Sciences Laboratory, [7]NOAA Global Monitoring Laboratory, [8]SRON Space Research Organisation Netherlands, [9]Carbon Mapper, Inc, [10]Department of Atmospheric and Oceanic Science / Cooperative Institute for Satellite Earth System Studies (CISESS), University of Maryland, [11]Energy Institute, Colorado State University, [12]Department of Systems Engineering, Colorado State University.

*Tai-Long He, Daniel J. Varon
Email: tlhe@mit.edu, dvaron@mit.edu

This PDF file includes:

Supporting text
Figures S1 to S15
Tables S1
Legends for Movie S1
SI References

Other supporting materials for this manuscript include the following:

Movie S1

Introduction.

This Supporting Information includes seven text sections, fifteen figures, one table, and one movie.

Text S1 describes the preparation work for the VLMR experiment.

Text S2 describes the lookup-table method used for the GOES ABI retrievals.

Texts S3-S5 provide quantification and retrieval methods for TROPOMI, VIIRS/SLSTR, and PACE-OCI.

Text S6 contains detailed information about the aircraft and truck-based in situ measurements and mass-balance calculations of source rates.

Text S7 discusses the comparison of GOES ABI scan modes and the sensitivity test on the moving-average window applied to GOES-18 mesoscale imagery and GOES-19 7-second imagery.

Figure S1 shows an example of forecast plume advection from HYSPLIT simulations used in planning the VLMR experiment.

Figure S2 shows the methane retrievals from Sentinel-3A and VIIRS overpasses over the release sites.

Figure S3 illustrates the instrumental spectral response functions associated with methane bands on the LEO satellites.

Figure S4 compares the PACE-OCI methane retrievals using the 1.6 μm and 2.13 μm reference bands.

Figure S5 summarizes the aircraft and the PUMAS mobile measurements around the two release sites and shows the NASA AVIRIS-3 retrieval of the test release prior to the main blowdown.

Figure S6 shows the angular wind direction geometry in the aircraft spiral mass-balance calculation.

Figure S7 shows the methodology for estimating the vertical layer thickness for a given aircraft loop in a given vertical profile.

Figure S8 shows the PUMAS mobile measurements of 3D winds and attenuated aerosol backscatter during the experiment.

Figure S9 shows a schematic of the PUMAS mobile lab driving path and time series of CH4 concentration measurements made from PUMAS.

Figure S10 illustrates the PUMAS mobile lab driving path and methane measurements, broken down into seven individual segments.

Figure S11 shows the saturation test on the Picarro gas analyzer.

Figure S12 compares in situ aircraft profiles with the corresponding GOES-19 ABI plume retrievals corresponding to the first two mass-balance estimates in Figure 3A in the main text.

Figure S13 shows a sensitivity test of GOES ABI IME and source-rate time series to the size of moving average windows.

Figure S14 shows the performance comparison between the five GOES ABI scan modes.

Figure S15 plots the sensitivity test on moving average window size for the GOES-18 mesoscale imagery.

Table S1 summarizes the average error statistics from the sensitivity test on delay time in calibrating aircraft measurements.

Movie S1 shows video footage of the methane plumes derived from GOES ABI.

Text S1: Preparation of the VLMR experiment.
Preparations for the VLMR gas pipeline blowdown experiment involved coordination across multiple NOAA laboratories and collaboration with the gas pipeline operator. Instrument deployment options were evaluated, including aircraft, mobile laboratory, and satellite observations. Site access, road conditions, and expected wind conditions were assessed to determine whether the mobile sampling platform could reliably intercept the plume. To support planning, HYSPLIT forecast simulations were performed in advance to predict plume transport and dispersion (e.g., Figure S1). HYSPLIT simulations were initiated approximately one week prior to the experiment using long-range weather forecast model results and were updated regularly as new meteorological forecast model results became available.

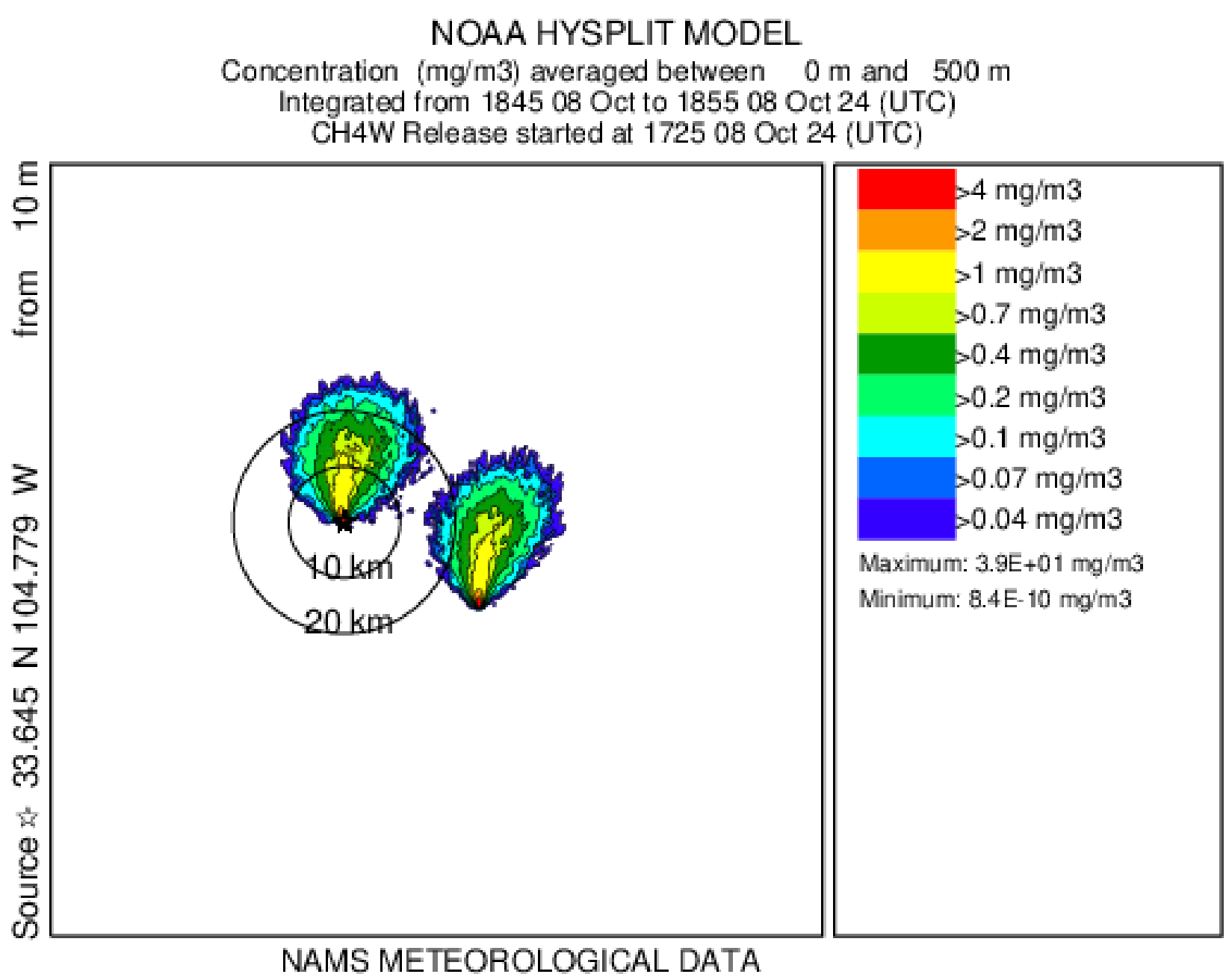

Figure S1. Example of HYSPLIT-simulated forecast concentrations, based on forecast meteorology, used for planning before the event started. This forecast graphic covers a 10-minute period from 1845-1855 UTC, which would occur during the 2nd hour of the actual event. The actual forecast graphic for field use included much more information (lat/longs, roads, etc.). The simplified version shown here does not include geolocation information, due to restrictions on the release of proprietary business information.

Text S2: GOES ABI retrieval and multi-band-multi-pass (MBMP) approach.
We simplify the GOES ABI retrieval algorithm that was originally based on the band-ratio approach of Varon et al. (2021). Rather than performing a full forward radiative transfer calculation for each pixel, we pre-compute a look-up table (LUT) that maps the observed band-averaged radiance ratio between the 1.6 μm and 2.25 μm ABI channels directly to methane column concentrations, and then correct for viewing geometry with a geometric air-mass factor (AMF). We assume a single US-Standard atmosphere, with background [CH_4] = 0.66 mol m^{-2} (~1900 ppb), [H_2O] = 790 mol m^{-2}, and [CO_2] = 143.3 mol m^{-2} (~400 ppm). We ignore vertical structure and also neglect CH_4 absorption in the 1.6 μm band (1–2 orders of magnitude weaker than at 2.25 μm).

The LUT is constructed by computing the top-of-atmosphere radiance in the 2.25 μm methane absorption window for a range of methane slant columns (-1 to +10 mol m^{-2}). For each methane slant column value, we use molecular absorption cross sections generated from HITRAN line parameters (via the HAPI Voigt line-shape model) for CH_4, H_2O, and CO_2 at the plume elevation (surface elevation + 0.5 km) pressure and temperature. We then weight the resulting transmittance by the Kurucz solar spectrum (Clough et al., 2005) across the ABI spectral window at 2.25 μm (2220–2270 nm) to compute the band-mean radiance ratio relative to the background (no CH_4 enhancement) case, and store this forward mapping between slant column and band-mean radiance ratio as the LUT. We then invert this relationship via interpolation, so that observed ABI radiance ratios can be directly mapped to methane slant column enhancement and subsequently converted to vertical column enhancement using the AMF.

For each resulting retrieval image (target), we construct a plume-free reference retrieval image from previous (pre-blowdown) scans and subtract it from the target to isolate methane plumes from surface artifacts. Using the operational 5- and 10-min scan modes, we construct a plume-free reference retrieval image from the average of seven cloud-free scans acquired during the same hour on a previous day when no plume was present, and subtract this reference image from the target retrieval to isolate methane plumes from surface artifacts. For the 30-s and 7-s mesoscale imagery without coverage from previous days, the reference image is instead defined as the mean methane image retrieved from 2 to 1 hours prior to the target scenes. We perform the retrievals and quantify total mass released and emission rate at the native 2-km resolution of the GOES ABI 2.25 μm band.

Text S3: TROPOMI quantification.
An estimate of the Integrated Mass Enhancement (IME) of both plumes at the time of the Sentinel-5P overpass was obtained from the operational TROPOMI CH_4 product (Copernicus Sentinel-5P, 2021).

The destriped total column methane data was used. A plume mask was created using a threshold with respect to the median methane concentration of the surrounding pixels (corresponding to a roughly 300km×300km scene with 710 pixels with successful retrievals), where the threshold (1930 ppb) was chosen to visually match the plume extent.

Methane concentrations were converted to enhancements by subtracting a reference concentration, computed as the median of the scene without the plumes. Subsequently, the enhancements were converted to per-pixel masses, using the surface pressure from the CH_4 product, the area of the footprint of the pixel, and the column averaging kernel of the methane retrieval.

For the column averaging kernel, the plume was assumed to be fully in the lowest layer of the TROPOMI retrieval (corresponding to the layer up to around 750 m above the surface). The IME was then computed by summing this value for all pixels in the plume. Uncertainties were computed by propagating the standard deviation of the concentration of the background pixels and the uncertainty of the CH_4 retrievals within the plumes as independent contributions.

Text S4: VIIRS/SLSTR retrieval and quantification
For VIIRS and SLSTR, methane enhancements were retrieved with a Multi-Band Multi-Pass (MBMP) method, slightly adapted from the one described by de Jong et al., 2025. Two adaptations were made, both improving internal consistency.

First, we used the following formula for the MBMP signal:

$$\Delta R_{MBMP} = \frac{c_{2.2}R_{2.2}}{R_{2.2'}} - \frac{c_{1.6}R_{1.6}}{R_{1.6'}} \text{ (Eq. S1)}$$

where the constants ci are the ratios of the median values of the band radiances on the reference day R_i' and on the measurement day R_i for the band with wavelength *i* µm. We then performed radiative transfer simulation to estimate methane concentration fields using ΔR_{MBMP}.

Second, an additional median subtraction was performed. The MBMP method yields methane enhancements with respect to an arbitrary reference concentration. To more accurately compute the enhancement in the plume, after finding the plume masks, the median of the pixels in the scene outside the plume masks was subtracted before computing the IME.

Otherwise, the same methods as in de Jong et al. (2025) were used, including destriping and resampling to a 500m-resolution grid of roughly 100 km × 100 km. The plume mask was also found using the same methods, by normalizing the enhancements with respect to the uncertainty and selecting areas with a deviation of more than 1.5 standard deviation and selecting only the two largest such areas per overpass. The uncertainty was similarly computed by summing the per-pixel uncertainties.

Figure S2 shows the resulting methane retrievals from Sentinel-3A and VIIRS over the western and eastern release locations on 8 October 2024. The 20:45 UTC NOAA-21 overpass is not included in Figure 2 in the main text because it falls outside the period of interest.

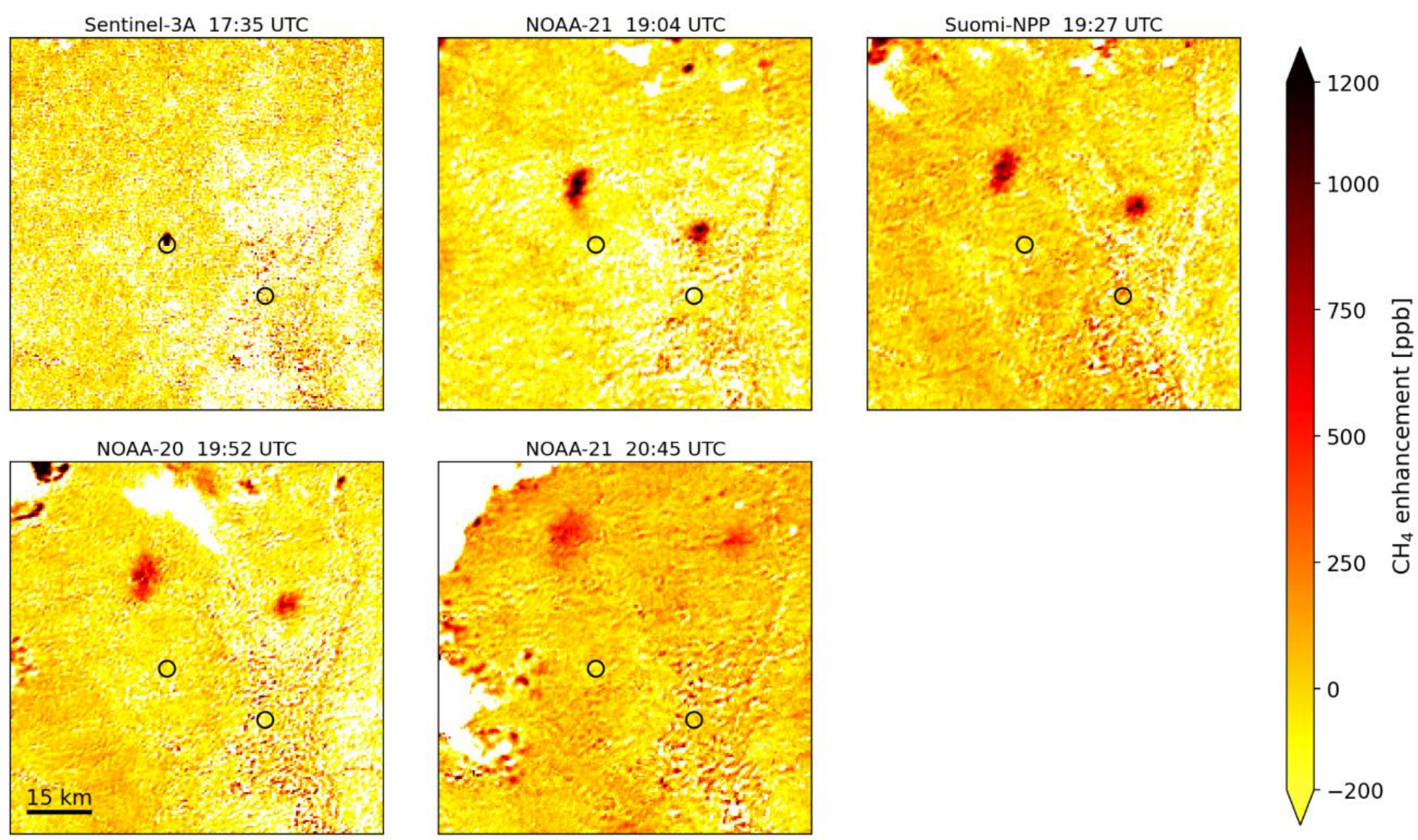

Figure S2. VIIRS and Sentinel-3A methane enhancement retrievals over the two VLMR release locations (black circles) on 8 October, 2024. White regions indicate retrievals masked by clouds. Retrieved total plume masses (sum of both plumes) are 104±26, 348±114, 352±109, 313±101, and 335±125 t, in chronological order.

Text S5: PACE-OCI retrieval

Methane enhancement retrievals were also performed using the Ocean Color Instrument (OCI) aboard the Plankton, Aerosol, Cloud, ocean Ecosystem (PACE) satellite, which launched on the 8th of February 2024. OCI has a nominal resolution of 1.2 km and has three SWIR bands relevant for methane retrievals, centered at 1.615, 2.130 and 2.260 µm respectively, as shown in Figure S3. The same retrieval method described in Text S4 was used, but using bands at 2.1 and 2.3 µm. Data was resampled to the same 500m-resolution grid used for the VIIRS retrievals

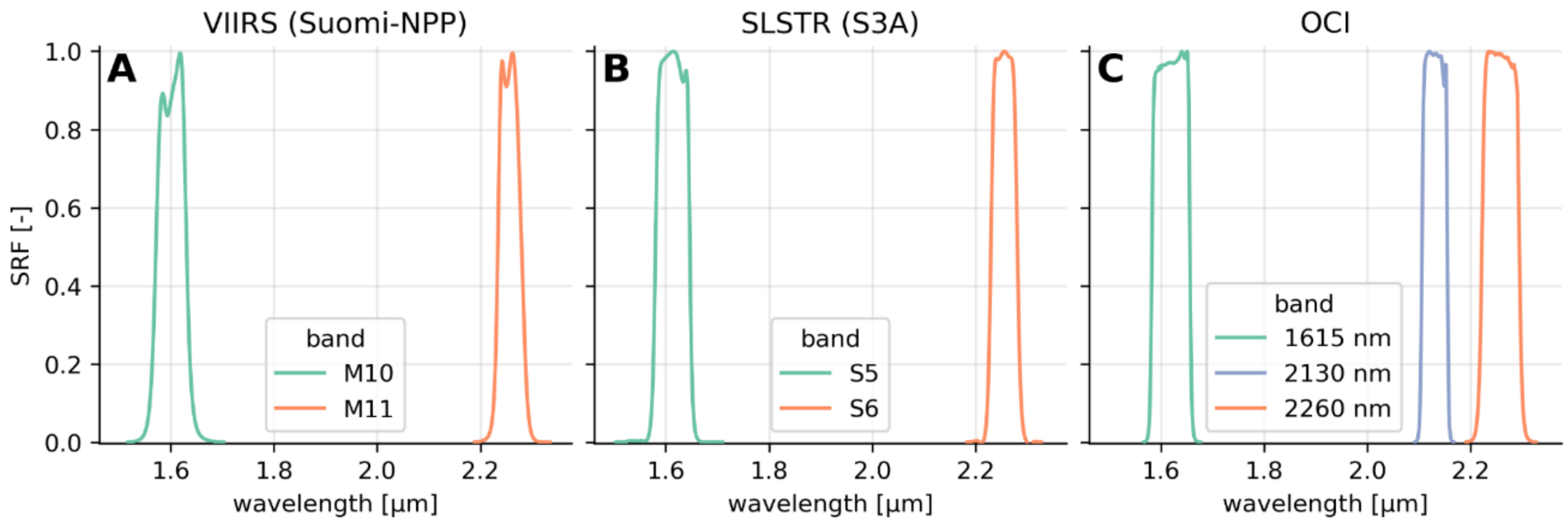

Figure S3. Instrument Spectral Response Functions (SRFs) of bands relevant to methane retrieval for (A) VIIRS on Suomi-NPP, (B) SLSTR on S3A and (C) OCI on PACE. SRFs for OCI were sourced from Meister et al. (2024).

As the 2.2 µm band is much closer to the µm 2.3 band compared to the usual 1.6 µm reference band used for other instruments, this combination is less sensitive to artifacts such as caused by differences in relative albedo of the surface, as illustrated in Figure S4. This smaller difference results in a cleaner retrieval. The OCI data was subsequently processed using the approach used for the VIIRS/SLSTR data as described in Text S5.

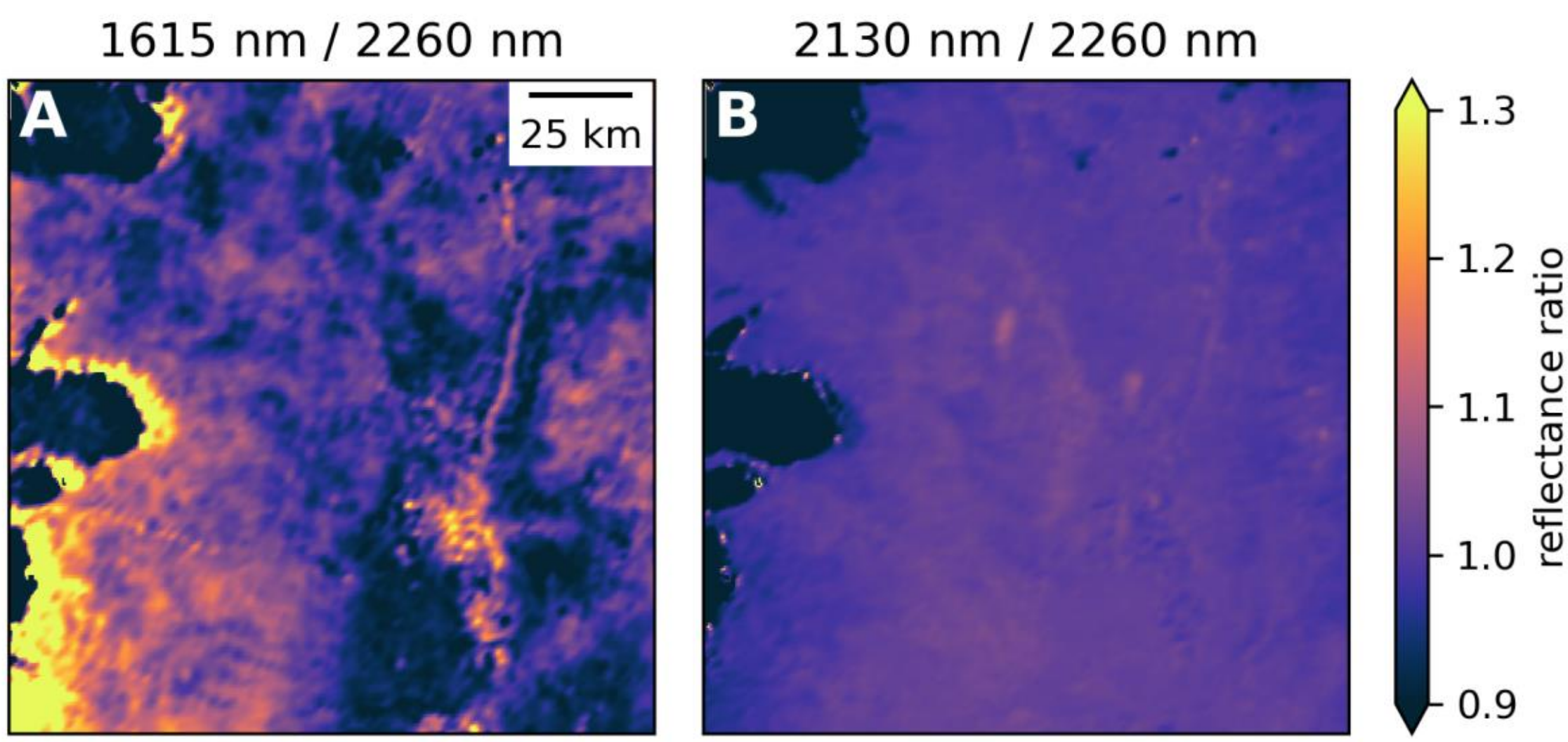

Figure S4. Ratios of reflectance measured in different SWIR bands of PACE-OCI for the overpass of the VLMR event. Comparing the usual 1.6 µm reference band (Standard Gain (SG) one used on PACE-OCI) and the 2.13 µm band to the 2.26 µm band, which has the strongest methane absorption. Shown are (A) 1615 nm SG over 2260 nm and (B) 2130 nm over 2260nm.

Text S6: In situ measurements and mass-balance calculation

As shown in Figure S5, the NOAA/ChampionX Mooney aircraft entered the experimental region at 16:20 UTC and initially flew to the western release site, where it remained in a holding pattern before the blowdown began. After the western release began at 17:22 UTC, the aircraft performed two near-source spiral profiles around the western source from 17:29 to 17:52 UTC, with loop altitudes of approximately 100–600 m agl. The maximum methane mixing ratio measured by the aircraft was 226 ppm at 17:45 UTC during western-source sampling. The aircraft then sampled the eastern source with two spiral profiles from 18:06 to 18:28 UTC at approximately 130–355 m agl. After completing the eastern profiles, the aircraft returned to the western plume for additional in-plume spirals to characterize plume vertical structure and

boundary-layer depth, followed by two cross-plume transects at 375 and 510 m agl approximately 25 km downwind of the western source. Mass-balance estimates of source rate from these transects (not shown in Fig. 3) are similar to the earliest spiral-based estimate (~200 t h^{-1}), but with much more uncertain timing because of the long travel distance. Aircraft measurements concluded at approximately 19:45 UTC.

NASA's Airborne Visible/Infrared Imaging Spectrometer-3 (AVIRIS-3), a high-spectral-resolution imager supporting methane point-source detection at 4.2 m spatial resolution, was also operating in the region at the time of the experiment. It was diverted from a nearby research flight track and captured methane enhancements from a test release at 16:13 UTC prior to blowdown at the eastern release site (Fig. S5B).

The NOAA PUMAS mobile laboratory sampled the eastern plume downwind of the release site. The truck measured a peak 1-s-average methane mixing ratio of 289 ppm at 17:46 UTC, approximately 1.85 km downwind of the eastern source and about 10 min after the blowdown began. It then moved to approximately 8.45 km downwind of the eastern source and conducted three crosswind transects. The first transect, from 18:20:29 to 18:33:31 UTC, measured a peak 1-min-average methane enhancement of 63 ppm but did not cover the full plume width and was therefore not used for the emission-rate estimate. The following two transects, from 18:35:30 to 18:45:29 UTC and from 19:08:30 to 19:19:31 UTC, measured peak 1-min-average methane enhancements of 10.1 ppm and 0.4 ppm, respectively. These two transects captured the full plume width and were used to estimate eastern-source emission rates by mass balance.

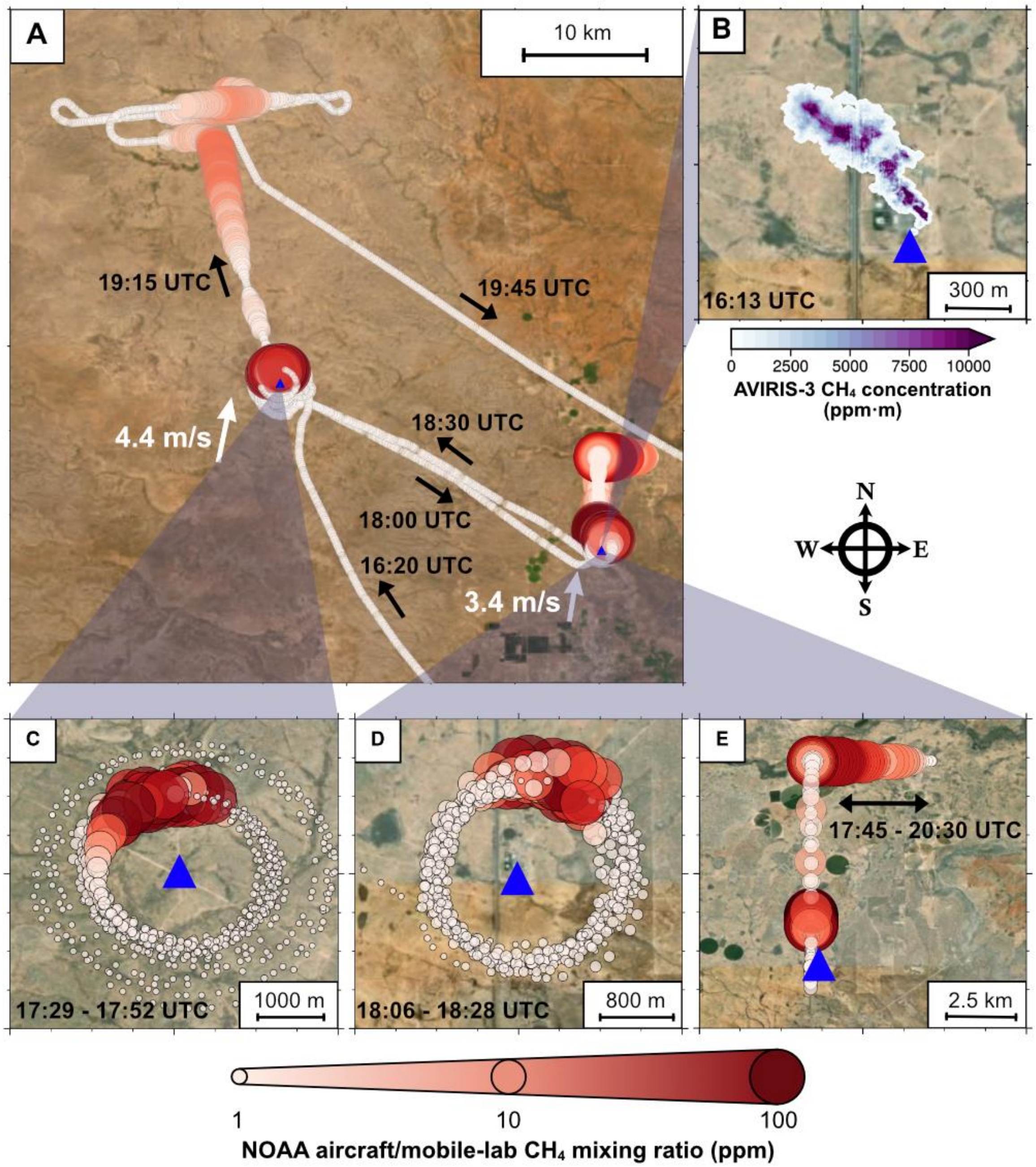

Figure S5. Aircraft and ground-based methane measurements during the VLMR experiment. (A) Methane mixing ratios (ppm) from the NOAA/ChampionX Mooney aircraft and PUMAS mobile laboratory. (B) AVIRIS-3 detection of the pre-blowdown test release. (C–D) Mooney spirals around the western (C) and eastern (D) release sites. (E) PUMAS plume transects near the eastern site. Blue triangle symbols mark the pipeline blowdown locations. Black arrows indicate direction of the Mooney aircraft and PUMAS mobile lab.

Text S6.1: Aircraft mass-balance analysis
Four wind treatments were used to estimate uncertainty in the aircraft mass-balance source rates for each loop of the NOAA Mooney spiral profiles:

1. Aircraft winds, loop mean: wind speed and direction from loop-mean 1-Hz aircraft GPS-based winds reported by Aspen Avionics.
2. Aircraft wind speed with plume-inferred direction: loop-mean aircraft wind speed combined with a concentration-weighted source-to-aircraft direction, representing the wind direction required to transport the plume from the source to the observed location.
3. HRRR winds: wind speed and direction from the NOAA HYSPLIT HRRR archive, interpolated to the source location and sampling time.
4. NAM winds: wind speed and direction from the NOAA HYSPLIT NAM archive, interpolated to the source location and sampling time.

HRRR is archived at 3-km horizontal resolution using hourly forecast cycles, whereas NAM is archived at 12-km resolution using 6-hourly forecast cycles.

The mass-balance methodology considers a cylindrical control surface around the emissions site. To estimate the mass flow rate through this cylindrical surface for a given loop, we sum the mass flow rate through the surface for each second during the loop, factoring in the estimated vertical thickness h of the layer that the loop is assumed to represent. For a given loop, the summation goes from the start time of the loop (0) to the end time of the loop (T):

$$\sum_0^T [C(t) - C_b(t)] \cdot \Delta w(t) \cdot \Delta h \cdot U_p(t) \cdot \beta(t) \quad \text{(Eq. S2)}$$

where

$C(t)$ (μmol CH_4 / mol air) = the second-by-second concentration of CH_4 measured during the loop by the Mooney aircraft;
$C_b(t)$ (μmol CH_4 / mol air) = the estimated background concentration of CH_4 during the loop;
$\Delta w(t)$ (m) = the second-by-second distance that the aircraft travelled during the loop;
Δh(m) = the estimated vertical thickness of the vertical layer associated with the loop;
$U_p(t)$ (m s^{-1}) = the second-by-second component of the horizontal wind velocity perpendicular to the direction of the aircraft at the location of the aircraft concentration measurement during that second (see Figure S4 below);
$\beta(t)$ [g m^{-3} / (μmol CH_4 / mol air)] = the second-by-second factor to convert from the volume/volume concentration measurements to mass/volume concentration values.

Since the control surface is a cylinder, we have used the orientation of the sampling site relative to the emissions source rather than the aircraft's heading relative to the ground, although the difference between the two is generally very small. It should be noted that the distance from the source varied during each loop, i.e., the cylinder's cross-section was not a circle. For each one-second measurement, the horizontal distance traveled was calculated as the radial distance for that measurement multiplied by the change in angle relative to the source location during that measurement.

Further, it should be noted that the elevation of each loop was not constant. For each loop, a concentration-weighted average elevation was used. This methodology ensures that the loop elevation used in the overall mass balance calculation reflects the measurements with strongly elevated CH_4 concentrations.

The mass flow rate for a given loop is then related to the time-resolved emissions rate from the source by subtracting an estimate of the travel-time from the source to the loop.

More details regarding the methodology are described below.

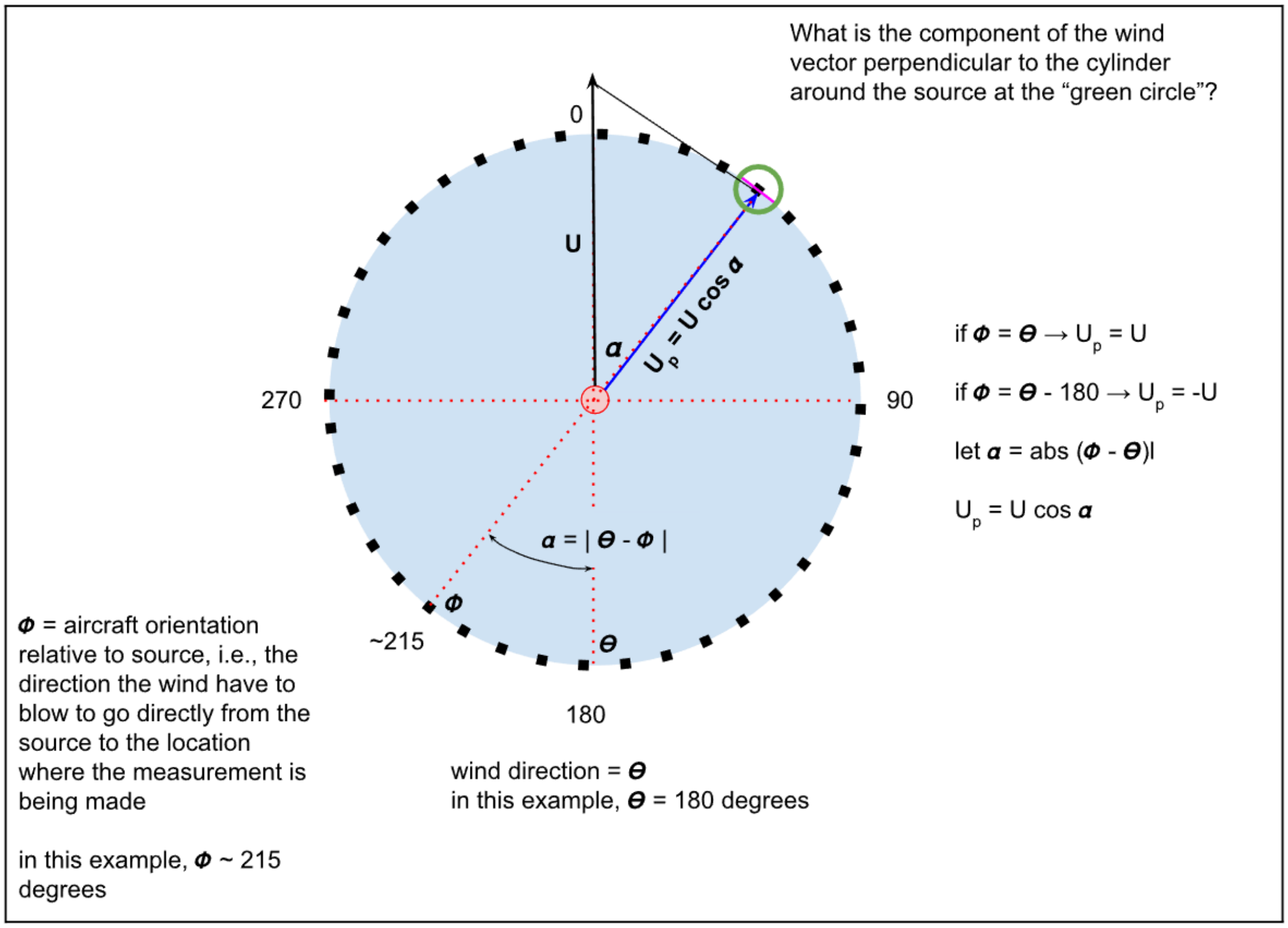

Figure S6. Angular wind direction factor in mass balance calculation.

Loop by Loop Analysis

Distinct loops were defined around the eastern and western source locations, in which the aircraft distance from the source was less than 2 km throughout the loop. The start of each East loop was assumed to be at an aircraft orientation relative to the source of ~58 degrees (the aircraft was approximately West-Southwest of the site). At that location, the wind direction would have had to be ~58 degrees for the wind to blow directly from the source location to the aircraft location. The start of each West loop was assumed to occur at a comparably defined orientation of ~325 degrees when the aircraft was Southeast of the West emissions site. This value ensured that the enhanced methane concentrations measured, at roughly 180 degrees, were well separated from the start and end of each loop. Also, the first loop around the West emissions site started at about 325 degrees.

The vertical layer thickness represented by a given loop in a given profile is assumed to be the difference in elevation from the midpoint between the loop and the next lowest-elevation loop and the midpoint between the loop and the next highest elevation loop in that profile. For the lowest elevation loop in a profile, the layer is assumed to extend to the surface. For the highest elevation loop, the layer is assumed to extend the same vertical distance above the loop as the difference in the loop elevation between the loop and the midpoint between the loop and the next lowest-elevation loop. This aspect of the methodology is shown schematically in Figure S7 below.

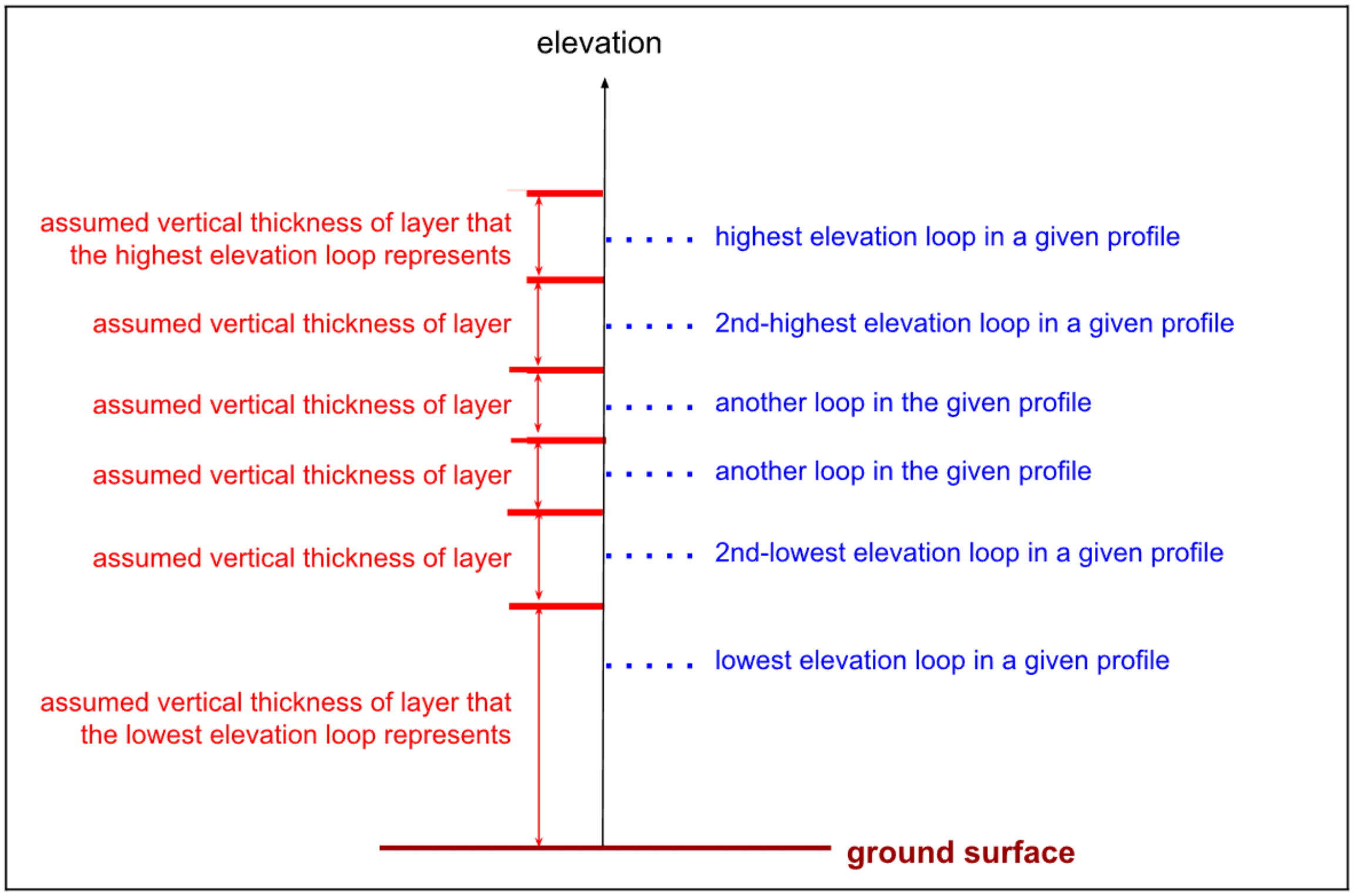

Figure S7. Vertical layer thickness estimation for a given loop in a given profile.

The aircraft GPS-associated measurements (lat, long, u, v, altitude, etc.) were delayed approximately 4-7 seconds due to data-processing and transfer constraints. The physical and chemical measurements (P, T, RH, CH4-ppbv, etc.) were delayed approximately 11 seconds primarily due to the inlet length. In the merged dataset from the aircraft flights, the physical and chemical measurements are automatically adjusted for the 11-second inlet-related delay. To match the GPS data to the physical-chemical measurement data, a specified number of seconds must be subtracted from each GPS timestamp's "UTC-Start" (seconds).

Numerical sensitivity experiments were conducted for GPS delays of 4, 5, 6, and 7 seconds to calibrate aircraft wind direction measurements. A statistical summary for the entire series of numerical experiments is shown below in Table S1. Based on these results, a GPS time lag of ~6 seconds was used as the "base" value in this analysis. However, the emissions rates estimated with time lags of 4-7 seconds are relatively consistent, and the sensitivity to this assumption is not large.

Table S1. Average error, absolute error, and RMSE of wind direction as a function of delay time.

Delays (s)	Ave error (deg)	Ave abs error (deg)	RMSE (deg)	Ave error (deg)	Ave abs error (deg)	RMSE (deg)
0	-23.9	23.9	90.8	-23.0	23.7	167.5
4	-10.3	10.4	45.3	-7.7	12.1	102.1
5	-7.0	8.0	35.7	-3.9	11.1	94.0
6	-3.6	6.8	28.2	-0.1	11.6	91.6
7	-0.2	6.0	24.7	3.6	12.1	94.7

Each loop took on the order of ~80 seconds to complete, and during each loop, CH_4 enhancements were measured at 15-20 seconds during the loops. The CH4 background for each aircraft loop was estimated as the 40th percentile of concentration values in that loop. Using other non-enhanced percentile values to characterize the non-enhanced "background concentrations" made only a trivial difference in the mass- balance emissions estimates and is not considered an important uncertainty in the mass- balance calculations.

The emission time for a given source differs from the aircraft measurement time because it takes time for the plume to travel from the source to the concentration measurement location. To estimate the uncertainty range in the travel time, we have estimated the minimum emissions time for a given profile by subtracting the maximum loop travel times (distance from source divided by the minimum loop wind speed (from methods 1, 2, 3, and 4)) from the time of the first loop in a given profile. The maximum emissions time for a given profile was estimated by subtracting the minimum loop travel times (distance from source divided by the maximum loop wind speed, from methods 1, 2, 3, and 4) from the time of the last loop in a given profile. The range in possible emissions times estimated in this way is used to estimate the uncertainty in the emissions time for any given profile.

Text S6.2: Mass balance analysis using NOAA PUMAS Mobile Lab
The PUMAS Doppler lidar operates at 1.54 µm, a wavelength at which atmospheric backscatter is dominated by aerosols rather than molecular scattering; signal-to-noise ratio therefore depends strongly on aerosol loading. The combined dynamics and aerosol information enables a robust boundary layer height retrieval based on mixing (e.g., w variance), wind shear, and aerosol gradient (Tucker et al., 2009). Figure S8 shows the wind and aerosol measurements from this experiment. The range resolution of both lidar channels was 62 m and the temporal resolution was 15 seconds for each profile of horizontal winds (i.e., one conical scan) and 1 second for the vertical wind channel. The wind speed shows a very consistent and well-defined low-level jet in the morning before sunrise, which decays as the daytime well-mixed boundary layer starts to grow. Boundary layer winds strengthened gradually from morning to midday. The boundary layer height grew substantially over the study period (17:36 - 19:00 UTC), making this measurement essential for accurate retrievals of methane emissions.

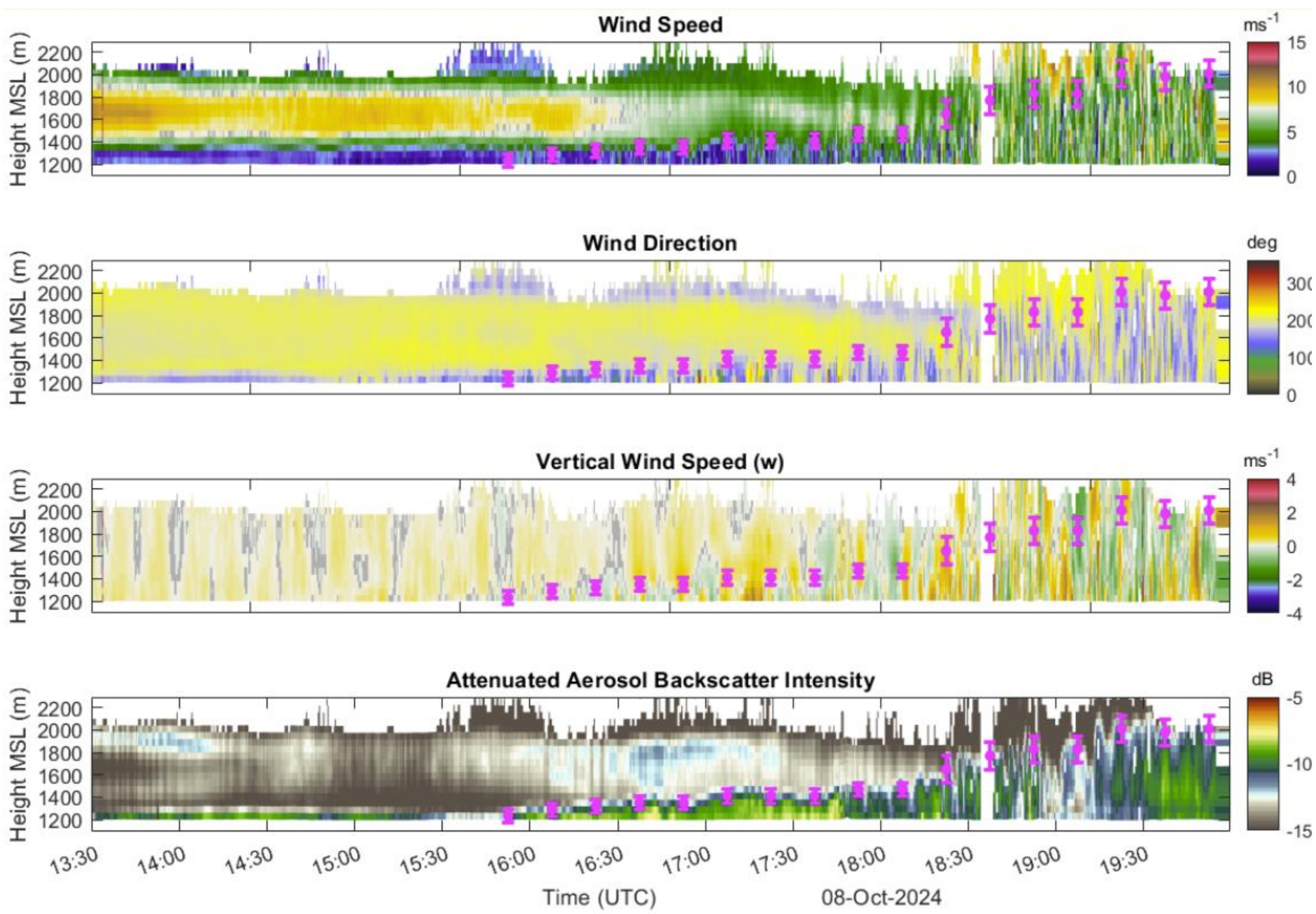

Figure S8. PUMAS lidar profile time series of (a) wind speed, (b) wind direction, (c) vertical wind, and (d) attenuated backscatter during the experiment. The pink points are boundary layer heights with uncertainty bars.

An Aeris instrument was deployed on PUMAS to measure methane and ethane at 1 Hz. The analyzer is a high-precision trace gas instrument based on mid-infrared (mid-IR) laser absorption spectroscopy for continuous, real-time measurements of methane and ethane (C_2H_6). By targeting fundamental vibrational absorption features in the mid-IR spectral region, the analyzer achieves high selectivity and sensitivity with low cross-interference from other atmospheric gases. The instrument offers sub-ppb detection limits, rapid response time (1 s), and stable operation suitable for both laboratory and field deployments. Its applications include urban air quality monitoring, natural gas leak detection, and characterization of methane sources, with ethane serving as a tracer to help distinguish fossil fuel emissions from biogenic methane. The analyzer was calibrated before and after the pipeline blowdown experiment. An online zeroing system was incorporated to zero the analyzer using ultra-zero air to track instrument drift during operation.

As shown in Figure S9, the PUMAS truck was near the eastern release site at the time of release, then, given the approximately southerly wind direction, PUMAS drove north with the goal of executing cross-plume transects far enough downwind that the methane would be well-mixed within the boundary layer. The first large plume intercept while driving north was incidental and did not transect the entire plume width, so it is not suitable for a representative emissions estimate. The later east-west legs are useful for emissions estimates, as the data shows that all or at least more than half of the plume was transected each time. The distance between the eastern release site and the east-west transects is ~8.5 km, which corresponds to ~28–35 minutes of transport time, given average winds of 4-5 m s^{-1} measured in the PBL. This is enough time for two eddy turnovers, and the observed plume widths are approximately three times the mixing height. Both are good indicators of the vertically well-mixed assumption.

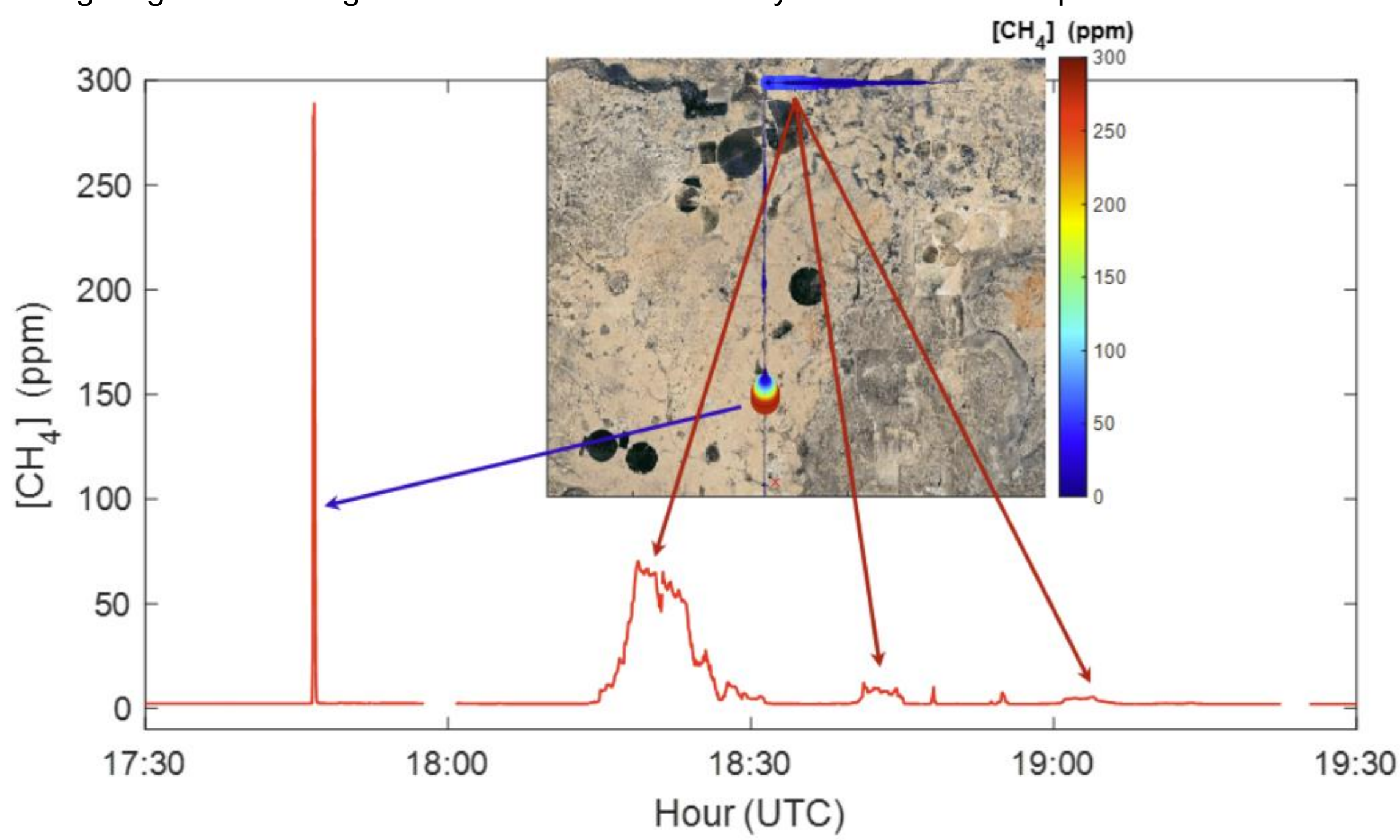

Figure S9. Time series of methane measured downwind of the eastern release facility during the pipeline blowdown experiment on Oct 8, 2024. The embedded map indicates the observation locations where methane enhancements were detected.

To apply mass-balance analysis to PUMAS measurements, we consider a plane (curtain) downwind of the emissions site and calculate the mass flow rate through this plane. We do this by summing the mass flow rate through the plane for each second during a given transect. In the following equation, for a given transect, each term is a function of time, and the summation goes from the start time of the transect (0) to the end time of the transect (T).

$$\sum_0^T [C(t) - C_b(t)] \cdot \Delta w(t) \cdot \Delta h \cdot U_p(t) \cdot \beta(t) \text{ (Eq. S3)}$$

where

C(t) (μmol CH_4 / mol air) = the second-by-second concentration of CH4 measured during the transect;

$C_b(t)$ (μmol CH_4 / mol air) = the second-by-second background concentration of CH4 during the transect;

Δw(t) (m) = the second-by-second distance that the mobile lab travelled during the transect;

Δh(m) = the second-by-second height of the plume at the location of the concentration measurement location during that second;

$U_p(t)$ (m s^{-1}) = the second-by-second component of the horizontal wind velocity perpendicular to the direction of the mobile lab at the location of the concentration measurement during that second;

β(t) [g m^{-3} / (μmol CH_4 / mol air)] = the second-by-second factor to convert from the volume/volume-based concentration measurements to mass/volume concentration values.

The mass flow rate for a given transect is then related to the emissions rate from the source by subtracting an estimate of the travel-time from the source to the transect, i.e., to account for the fact that the measurements occurred after the plume had traveled for some time downwind of the source.

We carried out an extensive series of numerical experiments and sensitivity analyses with respect to how the terms in Eq. S2 were calculated, and on the basis of this analysis, we estimate the overall uncertainty in the mass-balance flux estimates for each mobile transect to be ±25%.

As shown in Figure S10, the mobile-lab transects occurred along the east-west road about 9 km downwind of the source. The mobile lab driving path can be divided into 7 sections. The methane fluxes were calculated using Eq. S3, with h(t) set as the lidar-determined mixing height. We fit a seventh-order polynomial to h(t) in Figure S7 to time-interpolate a time-varying h across each transect. To get the boundary-layer averaged wind Up in Eq. S3, we vertically-average the wind profiles between ground and mixing height h(t), time-average nearby profiles (using a low-pass filter) to remove turbulent noise, then curve-fit to match their time-evolution with the 1 Hz methane measurements.

To estimate the travel time associated with truck-measured methane enhancements, we integrated the PUMAS wind backwards in time from the measurement time average, and determined when the integral reached the distance between the concentration-weighted transect location and the release point. This methodology may lead to an underestimate of the travel time because the plume likely did not follow a perfectly straight line from the source to the transect. This analysis gives an estimated travel time of ~30 minutes for the first transect from 18:20–18:30 UTC (red squares in Fig. S10), and ~26.5 minutes for the second transect from 18:35–18:45 UTC (green circles in Fig. S10).

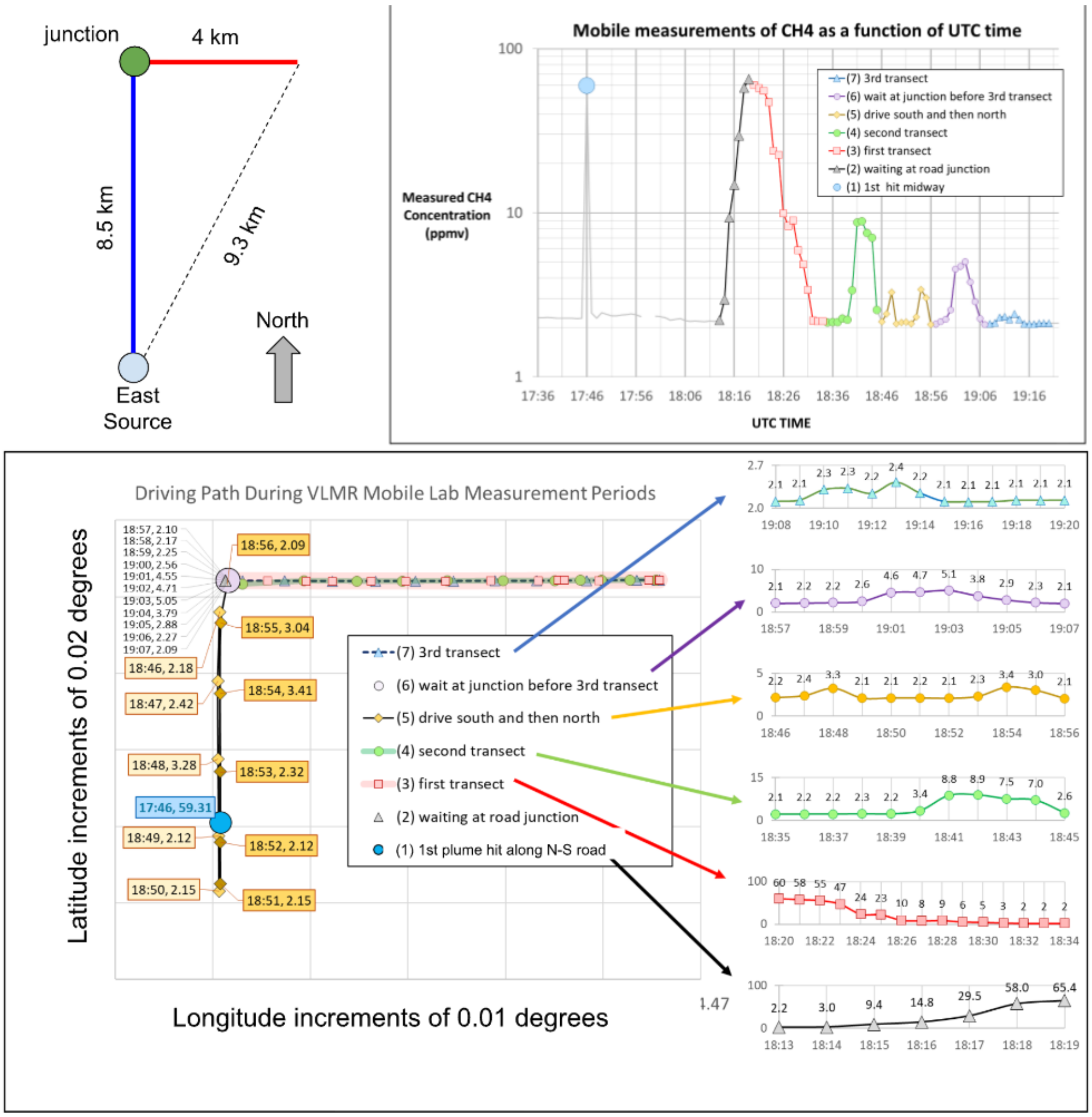

Figure S10. (Top) Schematic of the PUMAS mobile lab driving path and time series of CH_4 concentration measurements made from the mobile lab. (Bottom) Seven segments of the mobile lab driving path and associated CH_4 measurements.

Text S6.3: Diagnosing underestimated in situ mass-balance source rates

NOAA's Global Monitoring Laboratory installed a Picarro G2401-m aboard a ChampionX (formerly Scientific Aviation) Mooney aircraft for the National Observations of Greenhouse Gases using Aircraft Profiles (NOGAP) campaign beginning in mid-October 2024. The inlet was located in the wing, and approximately 2 m long, ~3 mm (⅛") O.D. stainless steel tubing with a flow rate of 255 sccm. Methane (CH4), carbon dioxide, carbon monoxide, and water vapor were reported every 2.5 seconds and interpolated to a 1 Hz time base. Additionally, ChampionX provided GPS and wind data calculated similar to Conley et al. (2017). The trace gas data time base has been shifted approximately 16 seconds for inlet and data transfer time lags to best align with the aircraft measurements. The Picarro instrument was calibrated immediately before and after the NOGAP campaign and run against a standard tank before and after each flight to determine instrument drift. We estimate uncertainties of ±2 ppb CH_4 relative to the WMO X2004A scale, and ±0.2 m s^{-1} for wind speed.

The Picarro G2401-m manual states the analyzer is linear up to 20 ppm CH4. To test the linearity of measurement above this threshold, the analyzer used during the October 8, 2024 flight was calibrated in the laboratory using a standard of 193.38 (±0.18) ppm CH4 in air. As shown in Fig. S11, the analyzer retrieved a value of 208.1 (± 1.2) ppm CH4. The highest value sampled on the October 8 flight was 225 ppm, and 25 plumes peaked above 49 ppm. We estimate an additional uncertainty of 2–8% for measurements between 49 and 225 ppm CH_4. We further note that when making these CH_4 measurements well outside the intended range of the instrument, the Picarro processing software takes approximately 8–10 seconds to report a value of this magnitude. This is significantly longer than the approximately 2–2.5 seconds between measurements when sampling background air. This delay in the measurement occurred both in the laboratory experiment and during the October 8 flight when the aircraft sampled the highest plume concentrations.

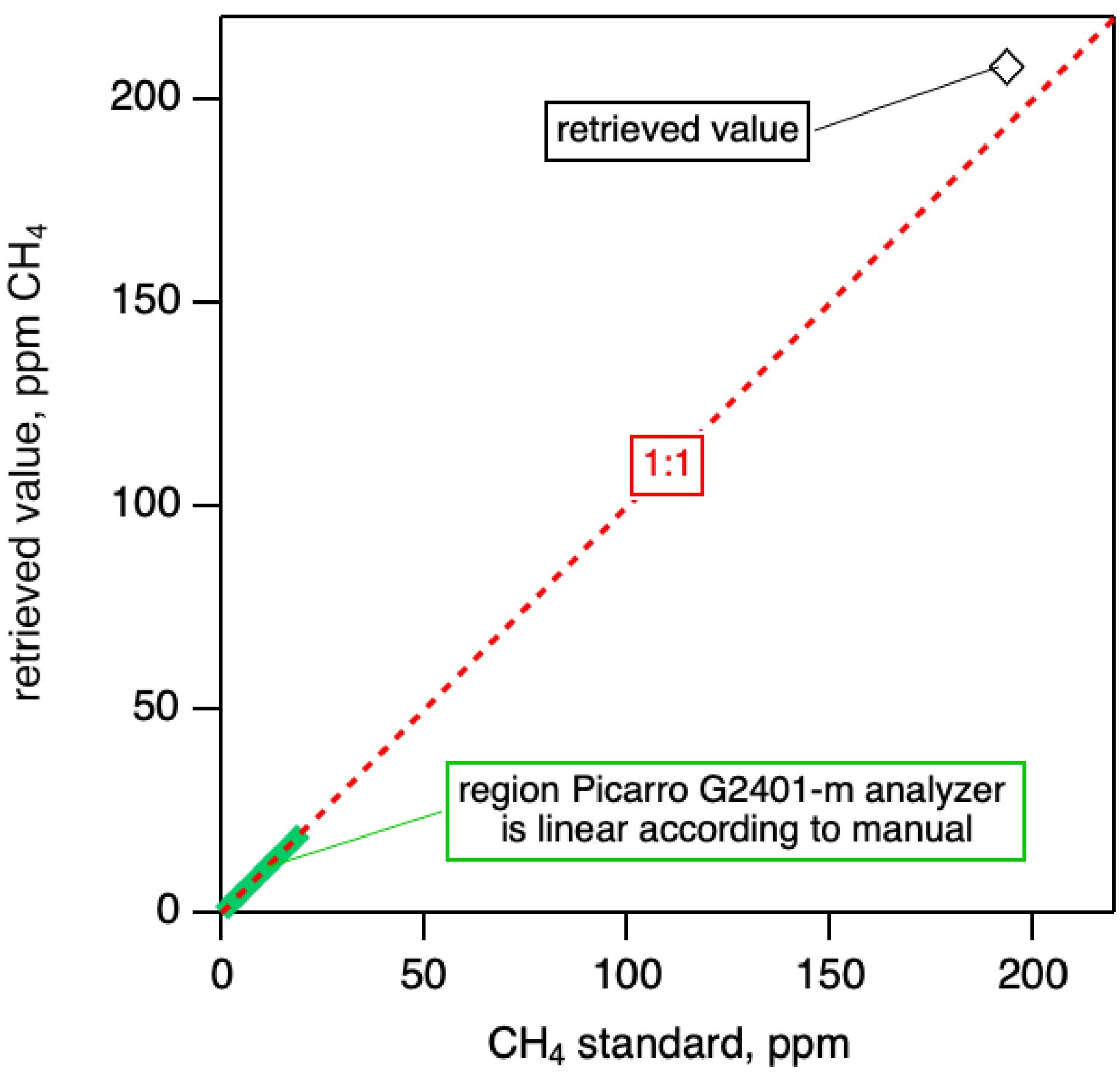

Figure S11. Laboratory results from the Picarro G2401-m analyzer sampling from a nominal 193 ppm CH4 in air standard. The region the manual states the analyzer has a linear response is highlighted in green.

Figure S12 provides a more detailed illustration of how this concentration-dependent measurement delay could contribute to an underestimation of the flux rates derived from in situ measurements. Figure S12A and B show the wind-rotated aircraft profiles over the western source, overlaid with the closest GOES-19 ABI 7-s methane retrievals, corresponding to the first two mass-balance estimates in Figure 3A. Several features could contribute to the underestimation of source rates from mass-balance analysis using the in situ measurements. First, the releases are highly transient, so no steady-state plume develops for in situ sampling. Second, the longer measurement time at elevated methane concentrations reduces the effective sampling frequency within high-concentration plume cores.

Figures S12C and D further demonstrate the crosswind cross-sections of aircraft measurements around the western source for the two aircraft spiral profiles. The two sampling profiles showed substantial spatial heterogeneity, with the highest methane enhancement represented by only a small number of measurements. This pattern is consistent with the longer measurement time of the Picarro analyzer at concentration levels outside its nominal measurement range, which reduced the effective sampling density within the plume core.

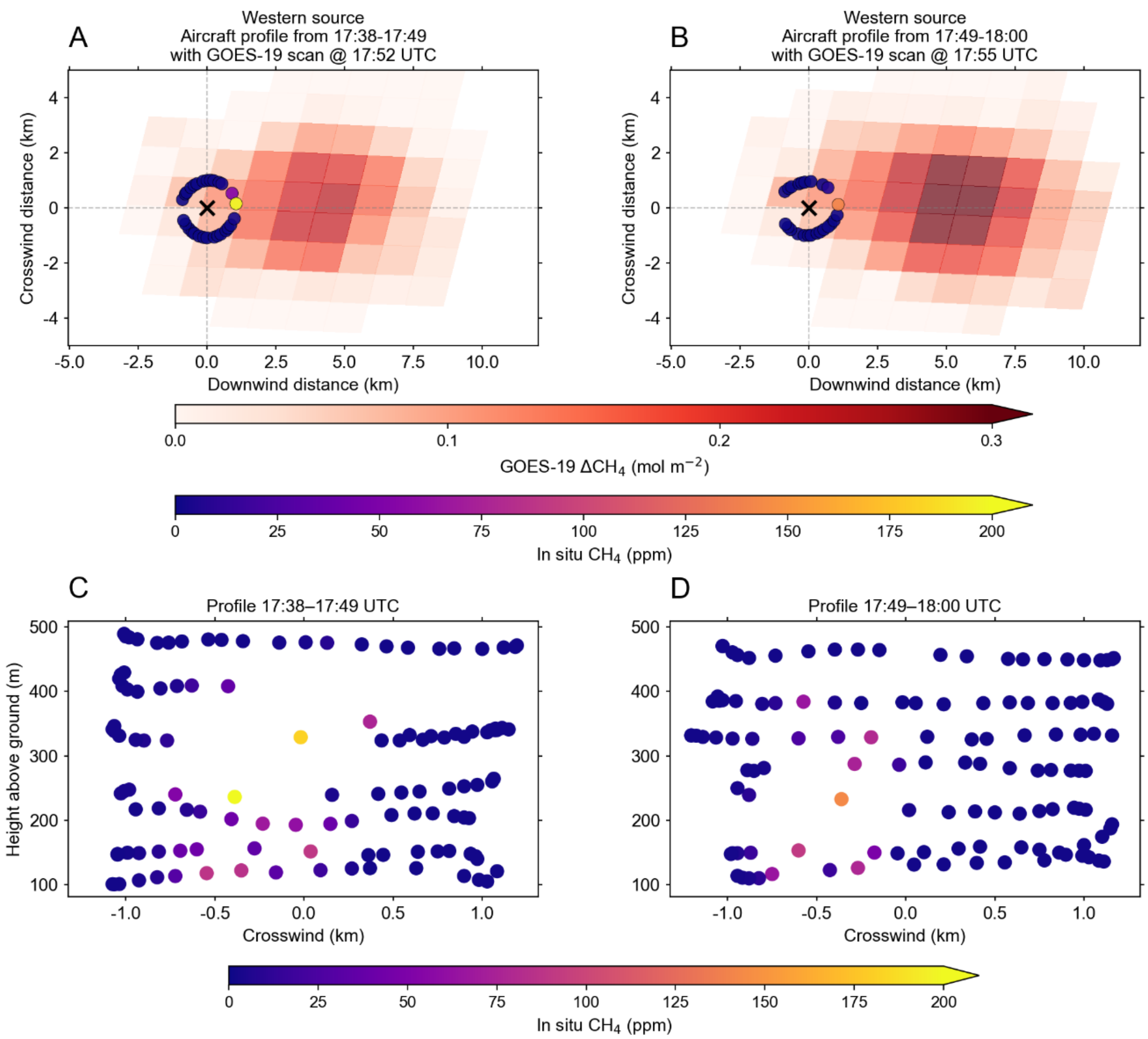

Figure S12. (A, B) Wind-rotated in situ measurements and corresponding GOES-19 ABI plume retrievals for the first two mass-balance estimates in Figure 3A. In situ measurements are shown as colored scatter points and GOES-19 ABI 7-second methane retrievals are shown as shaded fields. (C, D) Crosswind vertical cross-sections of aircraft measurements around the western source corresponding to the two spiral profiles.

Text S7: Comparison between GOES ABI scan modes and sensitivity test on moving averaging

Figure S13 shows the time series of IME and source rates from the raw retrievals and from moving averages calculated using 5-, 9-, and 15-min windows. The choice of averaging window has little effect on the IME results. For the western source-rate estimates, the differences among the averaging windows are also small, with differences of approximately 10% occurring only near the peak source rates. The eastern source-rate estimates are more sensitive to the averaging window: the 15-min moving average produces a substantially lower peak source rate, whereas

the 5-min moving averages are calculated from only three data points and are therefore less statistically robust. Overall, the 9-min moving-average window preserves the main temporal features of the source-rate time series without introducing apparent artifacts, and the ±1σ range provides a reasonable representation of the uncertainty associated with retrieval variability.

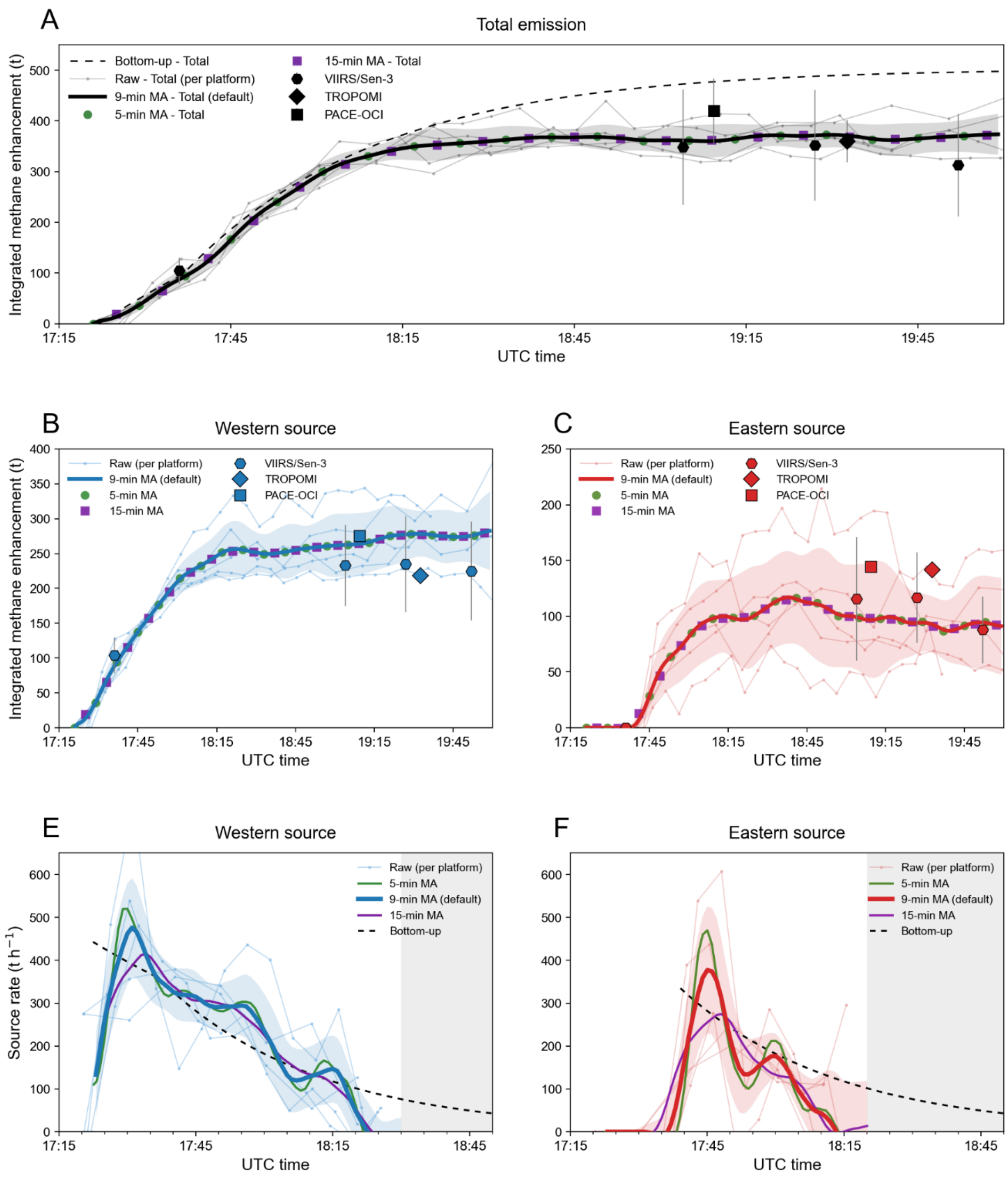

Figure S13. Sensitivity test of GOES ABI IME and source-rate time series to the size of moving average windows. (A-C) Time series of IME for total emissions, western source, and eastern source. Thin lines show the raw retrievals from each data stream; solid lines show the 9-min moving averages used in the main text; green circles show the 5-min moving averages; and purple squares show the 15-min moving averages. The markers with errorbars represent IME derived from LEO satellites. (E,F) Time series of source-rate estimates. Thin lines show the raw retrievals from each data stream; solid lines show the 9-min moving averages used in the main text; green lines show the 5-min moving averages; and purple lines show the 15-min moving averages. Dashed lines indicate the bottom-up model estimates.

Figure S14 compares the performance of different GOES ABI scan modes by calculating the signal-to-noise ratio (SNR) and the root mean square error (RMSE) for each scan. The SNR is defined as follows:

$$SNR = 10 \times \log_{10} \frac{P_{signal}}{P_{noise}} \text{ (Eq. S4)}$$

where P_{signal} is the average power of the isolated plume signal with non-plume pixels replaced by zeros and P_{noise} is the average power of background pixels (masking out plumes, water body, and clouds). Here we define the powers of imagery as

$$P_{signal} = \sum_{i,\ in\ plume} x_i \text{ and } P_{noise} = \sum_{i,\ in\ background} x_i \text{ (Eq. S5)}$$

A higher SNR indicates a stronger ability to isolate plume signals from the background, while a lower RMSE reflects greater precision in methane retrieval. The GOES-18 mesoscale imagery shows the best performance in both isolating plume signals and retrieving methane with high precision. The GOES-19 7-second imagery performs comparably to the GOES-18 and GOES-16 full-disk imagery, whereas the GOES-16 CONUS scans exhibit lower SNR and higher RMSE.

The smoothed GOES-18 mesoscale retrievals outperform all operational scan modes, increasing the SNR from -13 and -10 to values above -18 and reducing the RMSE by 50%, from 0.08 mol m^{-2} to below 0.04 mol m^{-2}. We also performed a sensitivity test on the choice of window size for smoothing the GOES-18 mesoscale retrievals. As shown in Figure S15, we find that both SNR and RMSE improve as the GOES-18 mesoscale imagery is averaged from 1-min to 2-min, and further to 5-min and 10-min windows. The best performance is achieved with 5-min and 10-min windows, which yield nearly identical results. For consistency, we adopt the 5-min window in the main text.

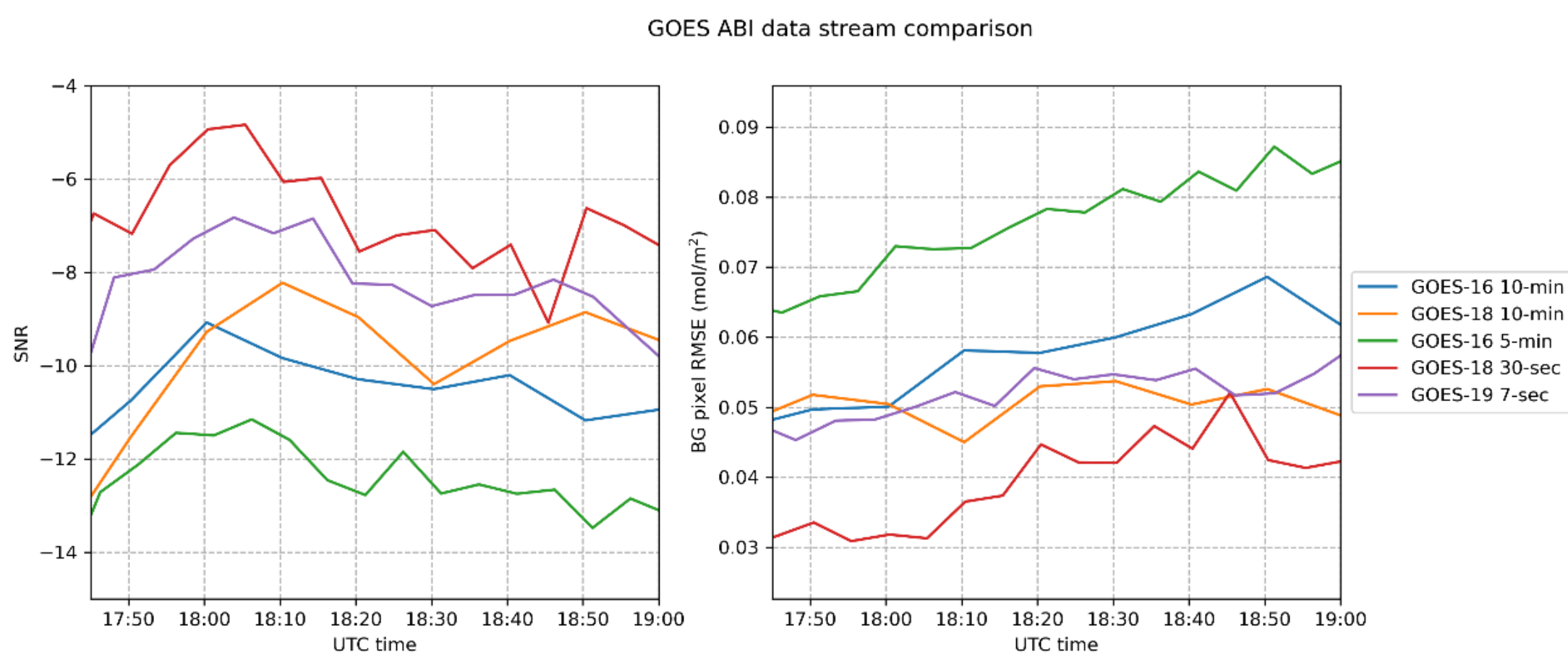

Figure S14. Time series of signal-to-noise ratio (SNR) and background pixel root mean square error (RMSE) for five GOES ABI data streams during the experiment.

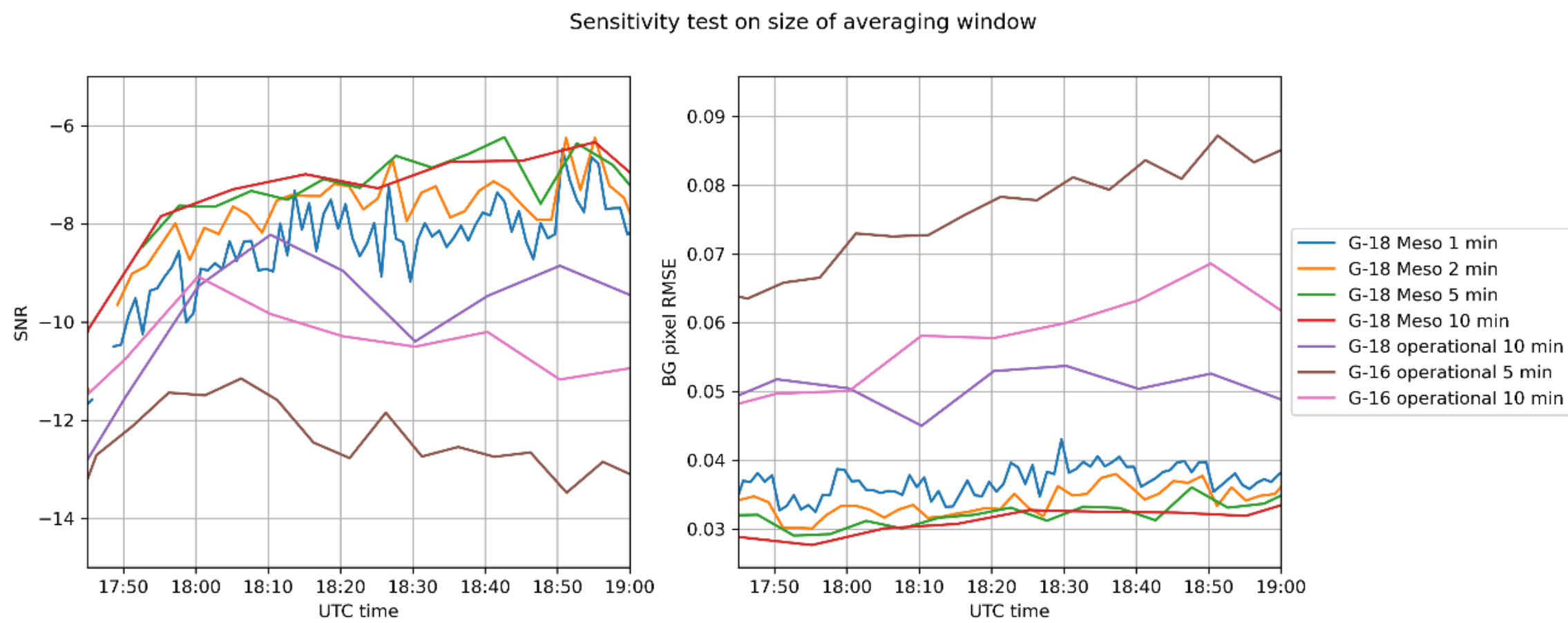

Figure S15. Time series of SNR and background pixel RMSE for GOES-18 mesoscale imagery smoothed using different window sizes, compared with operational GOES ABI data streams.

Movie S1 (separate file). Video footage of the methane plumes observed by GOES ABI.

SI References

1. Varon, D. J., Jervis, D., McKeever, J., Spence, I., Gains, D., and Jacob, D. J.: High-frequency monitoring of anomalous methane point sources with multispectral Sentinel-2 satellite observations, Atmos. Meas. Tech., 14, 2771–2785, https://doi.org/10.5194/amt-14-2771-2021, 2021.
2. Clough, M.W. Shephard, E.J. Mlawer, J.S. Delamere, M.J. Iacono, K. Cady-Pereira, S. Boukabara, P.D. Brown, Atmospheric radiative transfer modeling: a summary of the AER codes, Journal of Quantitative Spectroscopy and Radiative Transfer, Volume 91, Issue 2, 2005, Pages 233-244, ISSN 0022-4073, https://doi.org/10.1016/j.jqsrt.2004.05.058.
3. de Jong, T. A., Maasakkers, J. D., Irakulis-Loitxate, I., Randles, C. A., Tol, P., & Aben, I. (2025). Daily global methane super-emitter detection and source identification with sub-daily tracking. Geophysical Research Letters, 52, e2024GL111824. https://doi.org/10.1029/2024GL111824
4. Gerhard Meister, Joseph J. Knuble, Julia A. Barsi, Robert Bousquet, Leland H. Chemerys, Robert E. Eplee, Ulrik Gliese, Samuel Kitchen-McKinley, Shihyan Lee, Jeffrey W. McIntire, Frederick S. Patt, P. Jeremy Werdell, "On-orbit OCI characterization measurements from the first six months of the PACE mission," Proc. SPIE 13192, Sensors, Systems, and Next-Generation Satellites XXVIII, 131920A (20 November 2024); https://doi.org/10.1117/12.3033581
5. Tucker, S. C., C. J. Senff, A. M. Weickmann, W. A. Brewer, R. M. Banta, S. P. Sandberg, D. C. Law, and R. M. Hardesty, 2009: Doppler Lidar Estimation of Mixing Height Using Turbulence, Shear, and Aerosol Profiles. J. Atmos. Oceanic Technol., 26, 673–688, https://doi.org/10.1175/2008JTECHA1157.1.
6. Conley, S., Faloona, I., Mehrotra, S., Suard, M., Lenschow, D. H., Sweeney, C., Herndon, S., Schwietzke, S., Pétron, G., Pifer, J., Kort, E. A., and Schnell, R.: Application of Gauss's theorem to quantify localized surface emissions from airborne measurements of wind and trace gases, Atmos. Meas. Tech., 10, 3345–3358, https://doi.org/10.5194/amt-10-3345-2017, 2017.
